# The Categorisation of Non-Categorical Colours: A Novel Paradigm in Colour Perception

**DOI:** 10.1371/journal.pone.0059945

**Published:** 2013-03-25

**Authors:** Simon J. Cropper, Jessica G. S. Kvansakul, Daniel R. Little

**Affiliations:** Melbourne School of Psychological Sciences, University of Melbourne, Melbourne, Victoria, Australia; Dalhousie University, Canada

## Abstract

In this paper, we investigate a new paradigm for studying the development of the colour ‘signal’ by having observers discriminate and categorize the same set of controlled and calibrated cardinal coloured stimuli. Notably, in both tasks, each observer was free to decide whether two pairs of colors were the same or belonged to the same category. The use of the same stimulus set for both tasks provides, we argue, an incremental behavioural measure of colour processing from detection through discrimination to categorisation. The measured data spaces are different for the two tasks, and furthermore the categorisation data is unique to each observer. In addition, we develop a model which assumes that the principal difference between the tasks is the degree of similarity between the stimuli which has different constraints for the categorisation task compared to the discrimination task. This approach not only makes sense of the current (and associated) data but links the processes of discrimination and categorisation in a novel way and, by implication, expands upon the previous research linking categorisation to other tasks not limited to colour perception.

## Introduction

*“…the opponent-colours theory and the Young-Helmholtz three-colour theory could, with some modifications, very well exist side by side if one strictly distinguished between the process of excitation and the process of sensation and use the three colour theory for the former and my theory for the latter” (Ewald Hering, 1834–1918).*

Human colour vision has been simultaneously one of the most popular and productive, yet elusive and controversial areas of enquiry for well over a century [1]–[4]. It is one of the most fascinating areas of human research whether one examines this from a perspective of neurophysiology, psychology, philosophy, or the arts. The significance and generality of the work in colour vision is illustrated both by its longevity and by the regular occurrence of reviews, analysis and commentary related to different aspects of the issue from the neurosciences [5]–[7], through the cognitive sciences [8]–[14] to theoretical pattern recognition [15] and philosophy [16]. Each perspective contributes its own particular novel viewpoint, and each brings its own set of underlying assumptions to the area. Each of the cited authors acknowledge, from their own particular perspective, that the fundamental question of how we see colour not only remains unanswered, but is perhaps even less well defined than in its original form. It is also, notably, one of the few areas of research where no one viewpoint holds sway, and where the subjective and objective perspectives share much the same framework.

Categorisation and identification of stimuli has similarly been a mainstay of cognitive science research over the past decades [17]–[20]. The obvious link between these two areas is the observation that colour vision has been said to be categorical in nature; a common example of this being the perception of a rainbow as being split into categorically distinct hues. However, it is also self-evident that we perceive a continuum of hue and that to over-quantise this continuum, an underlying tenet of categorical perception [19], [21], is potentially counterproductive to the process of colour perception and vision more generally.

The current paper is concerned with furthering our understanding of these discrepant experiences by measuring and quantifying the link between the most basic of visual tasks: detection and discrimination of a coloured stimulus on the one hand, and the recognition and categorisation of that stimulus on the other. Theoretically our aim is to outline a consistent framework for understanding the relationship between discrimination and categorisation of visual stimuli, the time course of these two processes, and at what stage of processing the formation of a categorical stimulus representation i.e. being able to report identity, may come about. The work described here approaches this issue from a behavioural perspective with an examination of the relationship between discrimination and categorisation of stimuli which have a largely known effect upon early (detection) mechanisms in the visual pathway: cardinal chromatic stimuli [22].

### Cardinal stimuli and colour perception

The cardinal axes of colour space describe the chromatic properties of underlying opponent mechanisms instrumental in mediating the detection and discrimination of near-threshold chromatic stimuli. They only exist as independent behaviourally-orthogonal mechanisms at the threshold levels of stimulus contrast and as such form a specialised subset of psychophysically-defined mechanisms concerned more generally with the detection and discrimination of chromatic stimuli; by this we mean to say that the underlying properties of the mechanism(s) are defined through behavioural studies; the neural analogue (or mechanism) is far from clear ([22]–[25]; for specific reviews see [8], [9], [26]). In defining and articulating the properties of these cardinal stimuli, Krauskopf et al (1982) noted that the poles of the cardinal axes did not align with those of traditionally identified unique hues (e.g. red, green, blue & yellow) which, in turn, were thought to be a product of a specialised subset of psychophysically-defined mechanisms instrumental in the perception of colour [27]. Later work has taken this observation further and suggested that not only do cardinal axes fail to align with unique hue axes, but that the unique hue axes are not linear when plotted through the white point in cardinal space ([28]–[34]; for an excellent brief review see the introduction to Malkoc and Kingdom [35]. The lack of correspondence between these two sets of axes, cardinal and unique-hue, implies that the perception of unique hues cannot be explained by a simple linear transformation of the cone-opponent signal [34], [36], [37]. Providing a satisfactory explanation for this discrepancy between excitation (what we might predict from neural properties) and sensation (what we observe), as embodied in the quote by Hering at the head of this paper, persists as a fundamental question in colour vision, and indeed in vision more generally. Boynton's overview of this outstanding issue as a fundamental problem of colour vision remains one of the most cogent [38].

There have been several studies that have approached the issue of reconciling the opponent-cone processes with the psychological experience of unique hues using cone-modulating or cardinal stimuli for higher-level colour-perception tasks. These studies have shown that when observers are forced to make a decision about how ‘red’ or ‘green’ a cardinal (or cone-modulating) stimulus is, they are able to do so [22], [35], [39]–[43], even though the stimulus is not a good example of ‘red’ or ‘green’ in the sense traditionally implied by colour naming [27], [38], [44]–[48] being somewhat desaturated and perceptually more mixed than monochromatic light sources [35]. While colour naming categories can, therefore, be imposed on and plotted into a cardinal space, they do not fall out naturally from such relatively broadband stimuli and the basis of categorisation within this space remains controversial and not well defined. Nevertheless, there is evidence for enhanced sensitivity to colour (more precisely, binary-hue differences) close to a boundary defined by a line drawn in CIE XYZ colour-space between a unique yellow and unique blue [40], [49]. This enhanced sensitivity can be considered to be a form of a category boundary effect [19]. Hence, although cardinal stimuli generally do not provide good examples of learned colour names and are not usually used for the purpose of colour categorisation and naming, observers are able to categorise and identify these stimuli under two-alternative forced-choice conditions. However, it remains unclear whether the general increased sensitivity applies throughout the colour space when the number of colour categories is not limited to two and is instead potentially unbounded.

### Relationship between Categorisation and Discrimination

At a more general level, categorisation and discrimination might both involve similar underlying processes albeit with differences in the specific parameters involved in those processes. For one, there is evidence that people categorise colours based on their similarity to previously experienced colours [50]–[52]. This type of similarity-based judgement is also implicated in other types of tasks such as old-new recognition [53], [54], categorisation and inductive reasoning [55]–[57], and same-different discrimination [58]. Nosofsky et al. (2012) showed that the rather than representing fundamentally different tasks, categorisation and old-new recognition are best thought of as the same similarity-based process but with a lower threshold of evidence required for categorisation than for recognition. The same argument applies to the relationship between discrimination, which can be thought of as a single item recognition judgement when stimuli are presented sequentially, and categorisation; that is, discrimination capitalises on fine-grained differences between stimuli whereas even a coarse level of similarity might permit two stimuli to be judged as members of the same category.

The view that categorization and discrimination involve the same underlying processes sits in contrast to the view that discrimination is somehow altered by the presence of a category boundary. From a similarity-based perspective, optimal placement of category boundaries coincide with locations which are equally likely to belong to either of two categories (Ashby & Maddox, 1993); however, in the absence of other mechanisms like selective attention, which might act to perceptually stretch a relevant stimulus dimension along which a boundary falls, there is no a priori reason why discrimination would be improved at a category boundary.

On the other hand, it might be desirable for a system to have heightened sensitivity around a category boundary; however this enhanced sensitivity is likely to come after the initial stages of the stimulus processing. The boundary location is essentially an arbitrary judgement about a position along a stimulus dimension that has already been coded by the system; the location of that boundary being important for some subsequent (learned) processes but not the initial dimension-coding. This is particularly true in the case of colours, for which the relevant dimensions of hue, saturation and brightness are perceived holistically, are essentially co-dependent [51], [59]–[63] and on which selective attention is inefficient [50]. Nonetheless, enhanced sensitivity at category boundary locations has been demonstrated for Munsell colours [64], [65]. Whether this is the case for broadband cardinal colours is the focus of the current study.

### Colour, Categorisation and the Current Study

That colour perception is categorical has become somewhat of a common assertion more than an evidence-based observation. On the one hand we do see bands of colour in a rainbow, but we are also fully aware of gradual and consistent change of hue in the environment and even in the same rainbow upon careful examination. It is also the case that while categorical perception is inextricably linked to language and naming, it must be linked to perception free from language, as far as is possible, in order to be considered a truly perceptual quality. Non-verbal (i.e. not directed by learned colour names) categorical distinctions have been examined in the form of colour-sorting or ordering in perceptual colour space (e.g. [66]–[68] but the same non-verbal colour category distinctions have not been examined with stimuli more aligned to the early detection mechanisms. We adopt this as the starting point for the current study.

In the experiment described here, observers were presented with two coloured stimuli, one after the other, and required to decide whether they were identical (discrimination) or whether they were in the same (subjective) category (categorisation). In this way we are able to quantify task-driven differences in performance with a single comprehensive stimulus set; this, in turn, allows a single model to be applied to the data for both tasks and coherent link to be drawn between detection and categorisation of simple visual stimuli. This approach is slightly different from the norm, which typically only uses two categories and the observer has to judge between them. In our task there are no strictly enforced categories and the decisions are entirely up to the observers. This should minimise any learned selective attention processes since there is no objectively correct judgement to that the observer is trying to maximise. As an additional variable, we manipulate stimulus duration for the two tasks, since there have been suggestions that colour perception in particular should be affected by stimulus duration [43], [69]–[71] which facilitates contextualisation of our behavioural and theoretical approaches to some putative cortical properties.

## Methods

### Participants

Three observers (two males) with ages ranging from 19 to 25 participated in the current experiment. All had normal colour vision and normal or corrected-to-normal visual acuity. Each observer participated for approximately 60 hours in total, with each session typically lasting 30–40 minutes and consisting of four blocks of trials. All research was approved by the university's Human Research Ethics Committee. Participation was voluntary and contingent on the provision of written informed consent. Travel costs were reimbursed to participants after the experiment was over.

Observers were naive to the purposes of the experiment except for the obvious fact that we were measuring their discrimination and categorisation data spaces. As all trial blocks were randomly interleaved it was possible to examine the effect of experience to some degree; the boundaries of either space (discrimination and categorisation) remained unchanged over time. We examine the effect of learning in companion work in preparation.

### Apparatus and Stimuli

All stimuli were digitally-generated modulations of colour and luminance (when corrected for subjective factors - see below) and were displayed to a contrast-resolution of 14-bits per pixel by the TMS30c25 DSP chip on a VSG2/5 (Cambridge Research Systems) stimulus generator. The patterns were presented on a Sony G520 21” colour monitor with a mean luminance of 90 cd/m^2^ and CIE co-ordinates of the point of constant adaptation (whitepoint) calibrated to be (x = 0.333, y = 0.377). The monitor was driven at a frame rate of 75 Hz and a line rate of 52 kHz. All patterns were generated digitally during the line-flyback prior to presentation (i.e. there was no frame or line interleaving). The non-linear voltage-to-luminance relationship of the display was regularly measured using a photometric head (Graseby S351G) and was gamma-corrected using internal lookup tables on the VSG. The spectral properties of the monitor and calibration of the colour space were initially calculated from the individual monitor-phosphor outputs and the Smith/Pokorny cone fundamentals [72]–[74], correcting for the disparity between the vλ curve and Judd's revised short-wavelength sensitivity [75]. Day to day calibration was performed using the photometric head. The DACs were calibrated and checked occasionally and remained stable. The curve fitting procedure gave an R-value accounting for 0.998 of the variance. The total viewable display subtended a visual angle of 30 deg by 24 deg at the viewing distance of 0.5 m, with an effective pixel size of 0.036 deg by 0.036 deg. A small dark fixation point was located at the centre of the display. Viewing was conducted in a semi-darkened room (ambient light level approximately 10 cd/m^2^) and was binocular with natural pupils. No head restraint was used.

All stimuli were circular discs of subjectively equiluminant colour presented in the centre of a uniform grey field. The colour discs subtended 8 degs of visual angle, and were presented at 20-times detection threshold for each observer (see below) within a half-width raised cosine temporal envelope for a duration of 500 ms in the ‘long’ condition and 50 ms in the ‘short’ condition. The colours of the stimuli were drawn from the cardinal colour space described by Derrington, Krauskopf and Lennie (1984). The L and M polarities of the cardinal colour space were represented by a colour angle (the azimuth in the cardinal space) of 0 deg and 18 deg, respectively, while the –S and S points were represented by 90 deg and 270 deg respectively.

#### Subjective Equiluminance

The luminance angle (the elevation in the cardinal representation) of the minimal perceptual flicker of a 5 Hz counterphased grating, at a contrast of approximately 40-times detection threshold, was measured and taken as the subjective equiluminant point for each observer and each stimulus configuration: this setting was also checked regularly. The observers had to adjust the luminance angle until the flicker appeared minimal. The stimulus automatically refreshed to a new random luminance angle after 15 secs to avoid prolonged exposure and subsequent habituation being a confounding factor in the measurement. The final step size in the adjustment was 0.32 deg within the colour space and the mean of 10 estimates was taken for each stimulus. It was verified that both minimum motion and quadrature phase measures gave the same result as minimal flicker at our spatial and temporal frequencies [76], [77]. There was no significant difference between measures, in line with previous work [78]–[81].

#### Detection Thresholds

Detection thresholds for each stimulus were measured in a standard two-alternative forced-choice (2AFC) detection task. A stimulus was presented in one of two intervals and the observer indicated which interval the stimulus had appeared in by means of a mouse button-press. A feedback tone was provided. The contrast of the stimulus was adjusted according to the observer's response by a modified PEST staircase procedure [82] which gave a final threshold estimate of performance at the level of 75% correct. The contrasts of the stimuli used subsequently were scaled to the individual's detection threshold for each stimulus alone. Thus all stimuli were equidistant (in threshold multiples) from the neutral centre (white-point) of the colour space effectively scaling the space uniquely for each observer.

#### Categorisation and Discrimination

Stimuli for the categorisation and discrimination tasks were exactly the same; this is a critical and important feature of this study. The stimulus set consisted of colours distributed across the subjectively equiluminant plane of cardinal colour space, scaled to twenty times (20) an individual's detection threshold for that stimulus (colour) angle. There were a total of 220 different coloured stimuli giving a step of 1.6 deg between individual stimuli. Preliminary trials indicated this step-size to be of sufficient resolution while keeping the observing time to an acceptable level. The maximum angular resolution of the space was 0.35 deg (1024 subdivisions). The trial-blocks were set up so that each block spanned 17.6 deg of the equiluminant plane of cardinal colour space and consisted of 11 colours; 1 reference colour bisecting 10 test colours in angular space, resulting in 5 test stimuli either side of the reference stimulus in colour space. Thus, observers made a 360 deg sweep through colour space over the course of the experiment. Reference stimuli from each trial block were spaced 8 deg apart ensuring significant stimulus-overlap between neighbouring observing-blocks. This arrangement was critical for the calculation of category boundaries in the space as detailed below and has relevance of the influence of stimulus set on category boundary location [83]. Blocks were randomly interleaved throughout.

### Procedure

Once subjective equiluminance and detection thresholds had been measured, each participant completed both the categorisation and discrimination tasks interleaved in random order, with each data set being collected concurrently for each observer. Preliminary observation showed there to be no effect of trial order.

In discrimination trial-blocks, the observer was required to indicate, by means of a mouse button-press, whether the two stimuli appeared to be physically identical or not. In the categorisation trial-blocks, the observer was required to indicate whether they would choose to put the stimuli in the same category or not. No definition of ‘category’ was given to the observers, rather they were encouraged to use their own definition; a deliberate decision upon in order to minimise the influence of learned colour names. Across all trials, in each block, test stimuli were presented followed by the reference stimulus for that block. Test stimuli were presented in a randomised order and included any of the possible colours from the block; giving 11 possible different colour pairs (one test plus reference in that order). In all cases the inter-stimulus-interval between test and reference was 0.67 seconds (50 frames). The presentation and arrangement of stimuli was identical for either task apart from the random ordering of pairs within each block. For each of the discrimination and categorisation tasks, all 20 blocks were shown and repeated four times each, with two practice blocks being presented at the start for each observer. This gave 40 (4×10) trials per data point for each of the two tasks.

### Data analysis

Data from both tasks, categorisation and discrimination, were treated in exactly the same way. Because each task is a yes-no judgement, the raw responses from each observer were transformed into d′ values to give a bias-free estimate of performance [84], [85].

For each block, a d′ value was calculated for the different colour stimuli pairs producing ten d′ values for each individual block (40 trials per d′ value). The five d′ values either side of the central d′ value represent measures of observer sensitivity (or judgement of a difference in category) as the test moved away from the reference in distance in colour space. The d′ value was then transformed back into an unbiased proportion of ‘same’ for each stimulus pair for clarity, and this value then used in the figures and modeling.

Unlike the discrimination condition, in the categorization task there is not necessarily a correct or incorrect response; nonetheless, for consistency we treat the categorisation data as if it were the same as the discrimination data for the purpose of computing d′. Thus the ‘hit’, ‘miss’, ‘false alarm’ and ‘correct reject’ were defined in the same way for both tasks. The fundamental characteristics of the data were not changed by this approach, indeed there was minimal impact upon the data overall (well within the error estimate of the raw data) but we felt to be the appropriate course of action.

### Estimation of boundaries in the two data spaces

A common and clear way of presenting discrimination data is to use a measure of Just Noticeable Difference (JND); for consistency we have adopted this approach for both the discrimination and the categorization data. As described above, for each block a d′ value of performance was calculated for each of the stimulus pairs indicating performance on the discrimination task. To calculate the difference in colour angle (either side of the central reference) required to give a performance of 80% ‘different’ the measured values were fitted with a cumulative Weibull curve. The values of this curve-fit were then used to calculate the 80% (d′ = 1.68) JND threshold either side of the reference colour. Given each reference stimulus was judged the same or different from stimuli either side of its location in colour space, two initial 80% JND thresholds emerged for each region.

**Figure 1:**
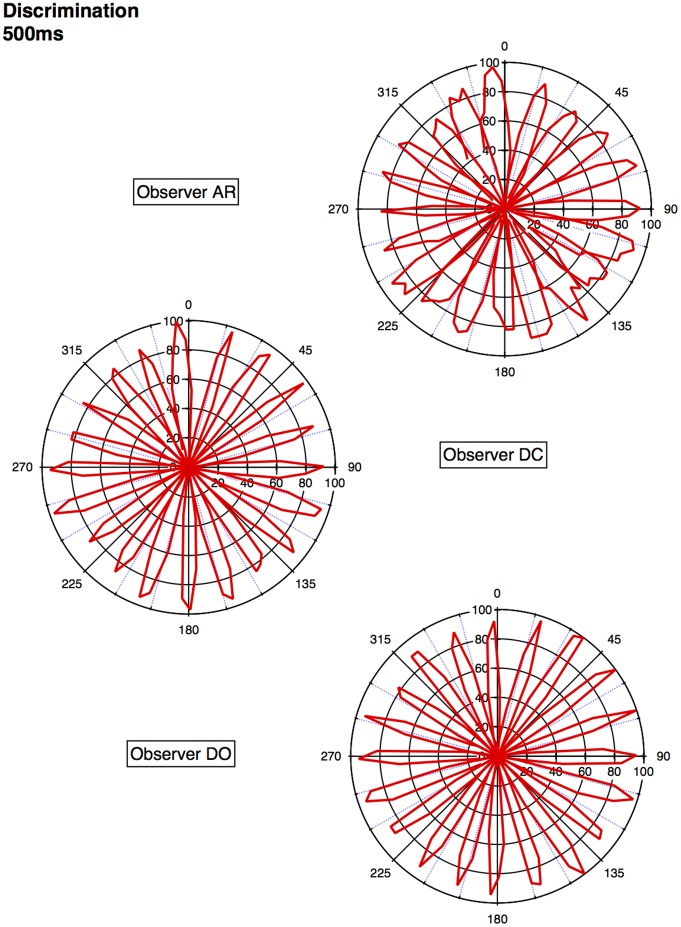
Polar plots showing performance in the long duration (500 ms) discrimination condition. Radial coordinates give the colour angle of the test stimulus, axial coordinates the unbiased proportion considered ‘same’ (see text). Reference stimuli are located at the central colour angle of each ‘petal’ which also coincides with the apex of the petal.

**Figure 2:**
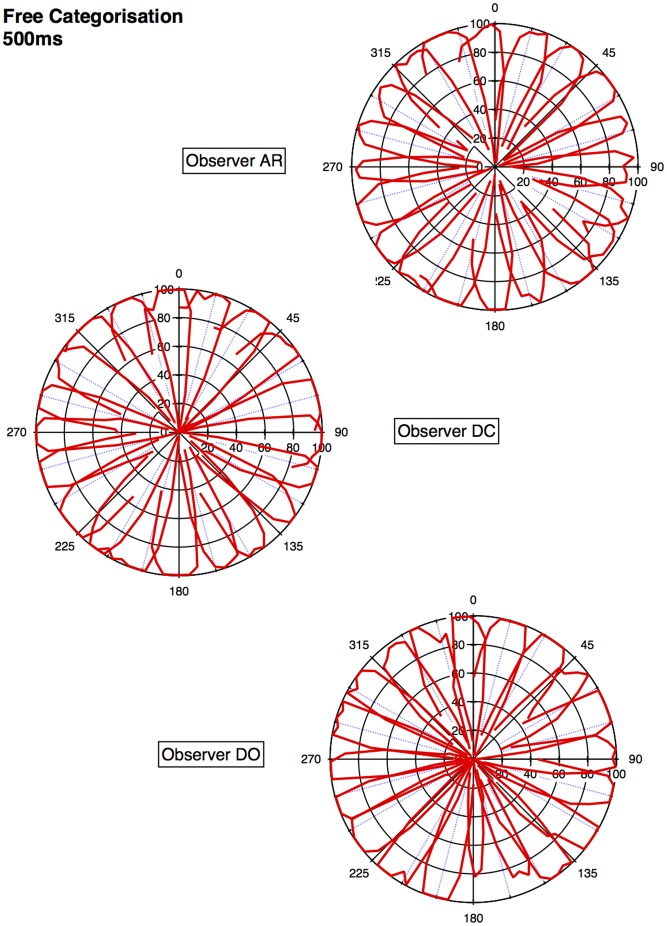
Polar plots showing performance in the long duration (500 ms) categorisation condition. Radial coordinates give the colour angle of the test stimulus, axial coordinates the unbiased proportion considered ‘same’ (see text). Reference stimuli are located at the central colour angle of each ‘petal’ which also coincides with the apex of the petal.

**Figure 3:**
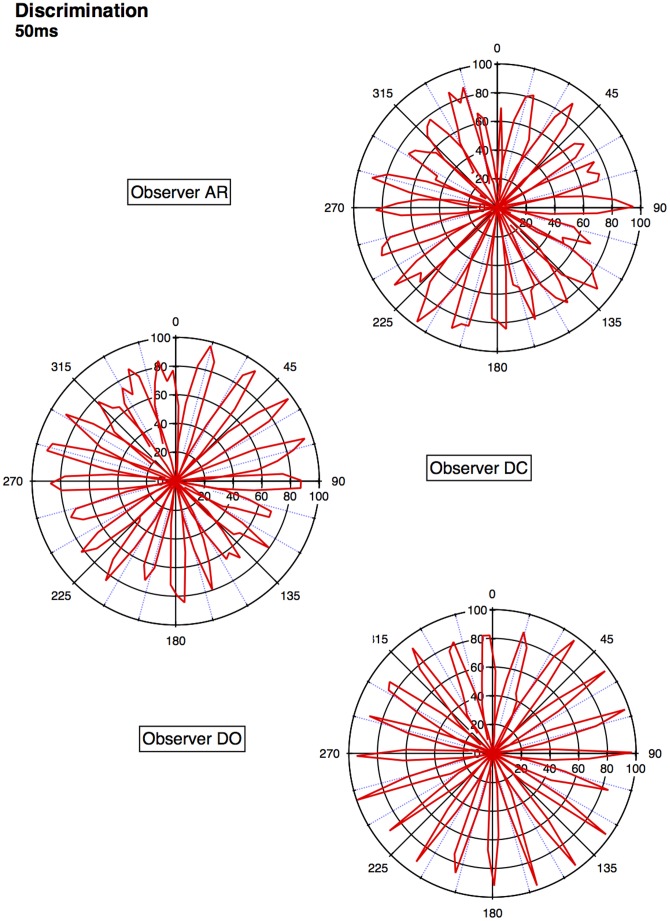
Polar plots showing performance in the short duration (50 ms) discrimination condition. Radial coordinates give the colour angle of the test stimulus, axial coordinates the unbiased proportion considered ‘same’ (see text). Reference stimuli are located at the central colour angle of each ‘petal’ which also coincides with the apex of the petal.

**Figure 4:**
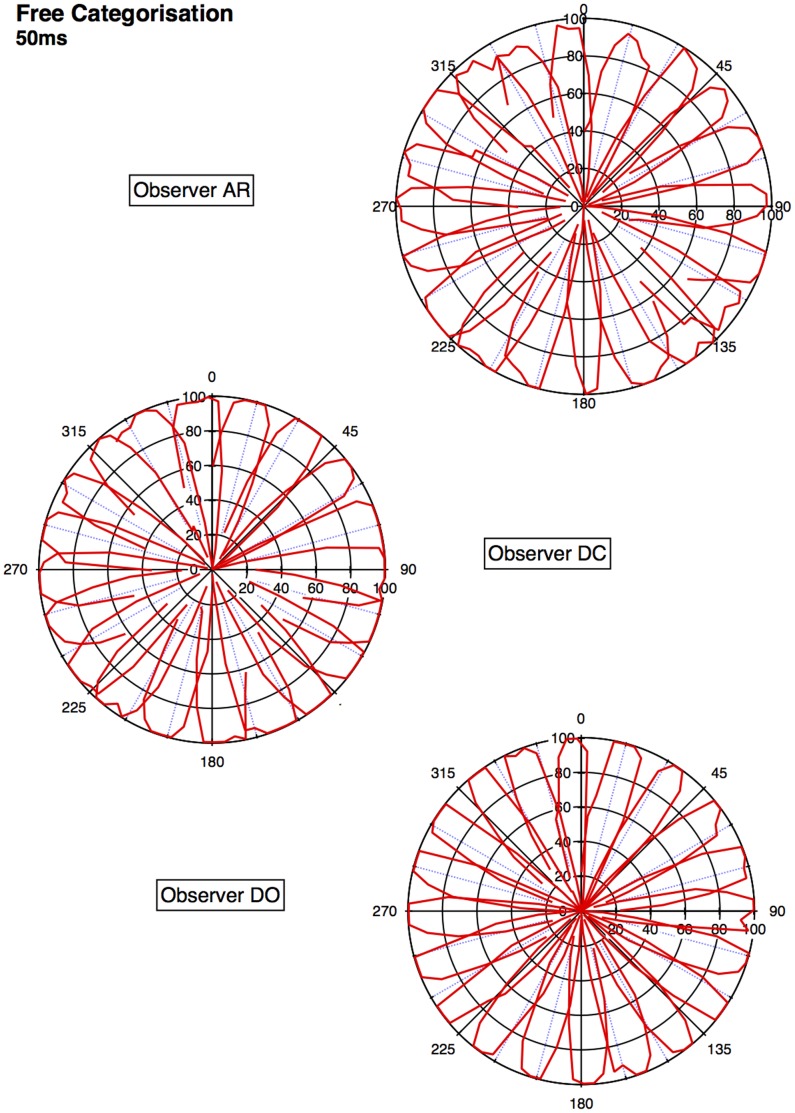
Polar plots showing performance in the long duration (50 ms) categorisation condition. Radial coordinates give the colour angle of the test stimulus, axial coordinates the unbiased proportion considered ‘same’ (see text). Reference stimuli are located at the central colour angle of each ‘petal’ which also coincides with the apex of the petal.

An approximate category boundary was defined as the colour angle (x-axis) where two categorisation curves intersected below a value (proportion ‘same’ category) of 0.8. Some category boundaries are more definitive than others, depending on exactly where the curves cross on the y-axis. This is realised visually in the polar plots (Figures 1, 2, 3, 4) by the extent to which each curve forms a distinct ‘petal’.

Each of these two methods take a conservative criterion on both tasks (80% correct in discrimination; 80% of the time considered to be in the same category), facilitating the utility of comparison within-subjects between the two tasks. Similar treatment for all observers facilitates between-subject comparisons.

We are mindful that to some degree these are arbitrary definitions; however, in conjunction with the full data sets and the modeling they are nonetheless a useful representation

## Empirical Results

**Figure 5:**
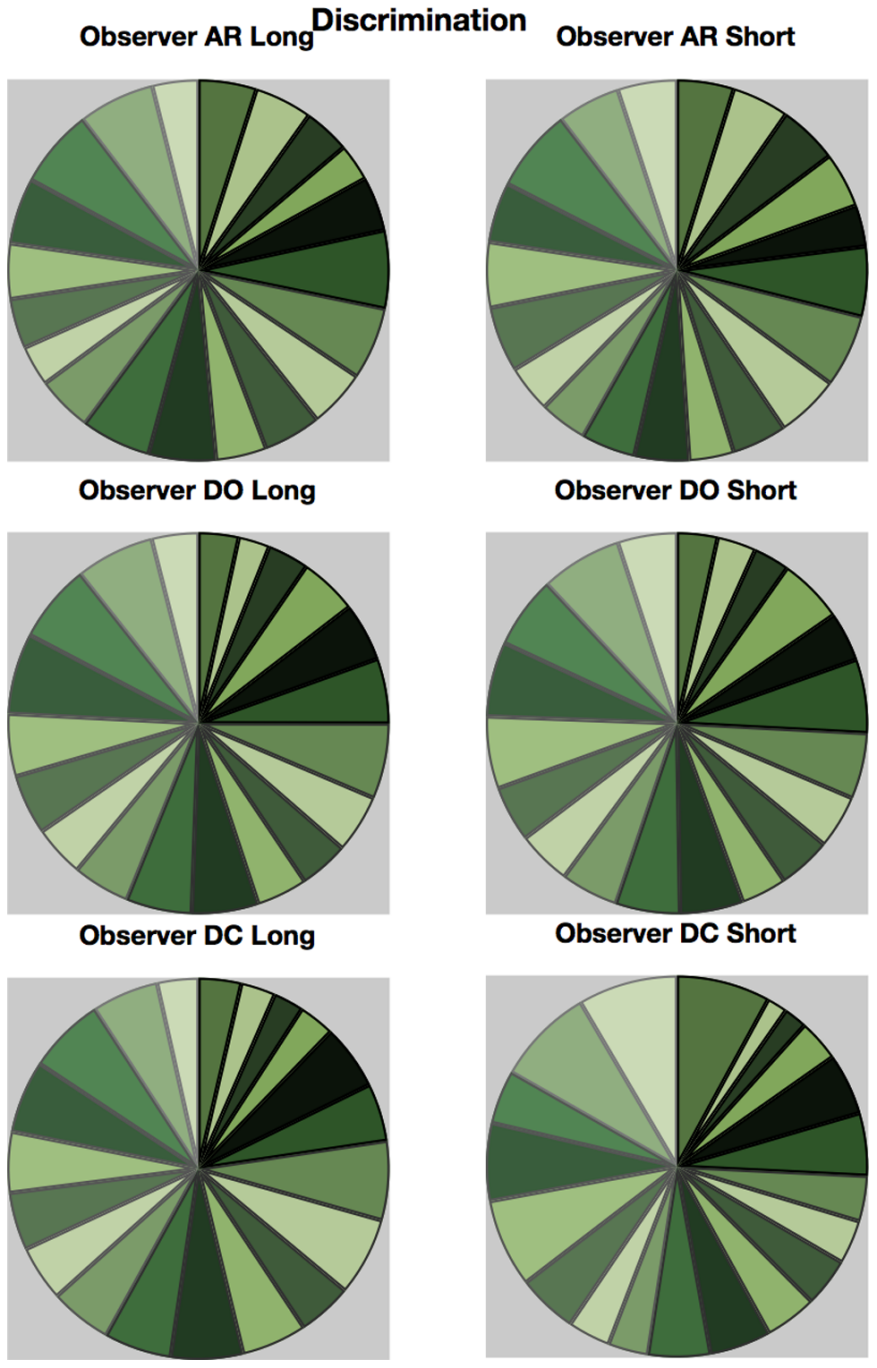
Just Noticeable Differences (JNDs) for the discrimination task arranged by observer (rows) and duration (columns). See text for details of calculation.

**Figure 6:**
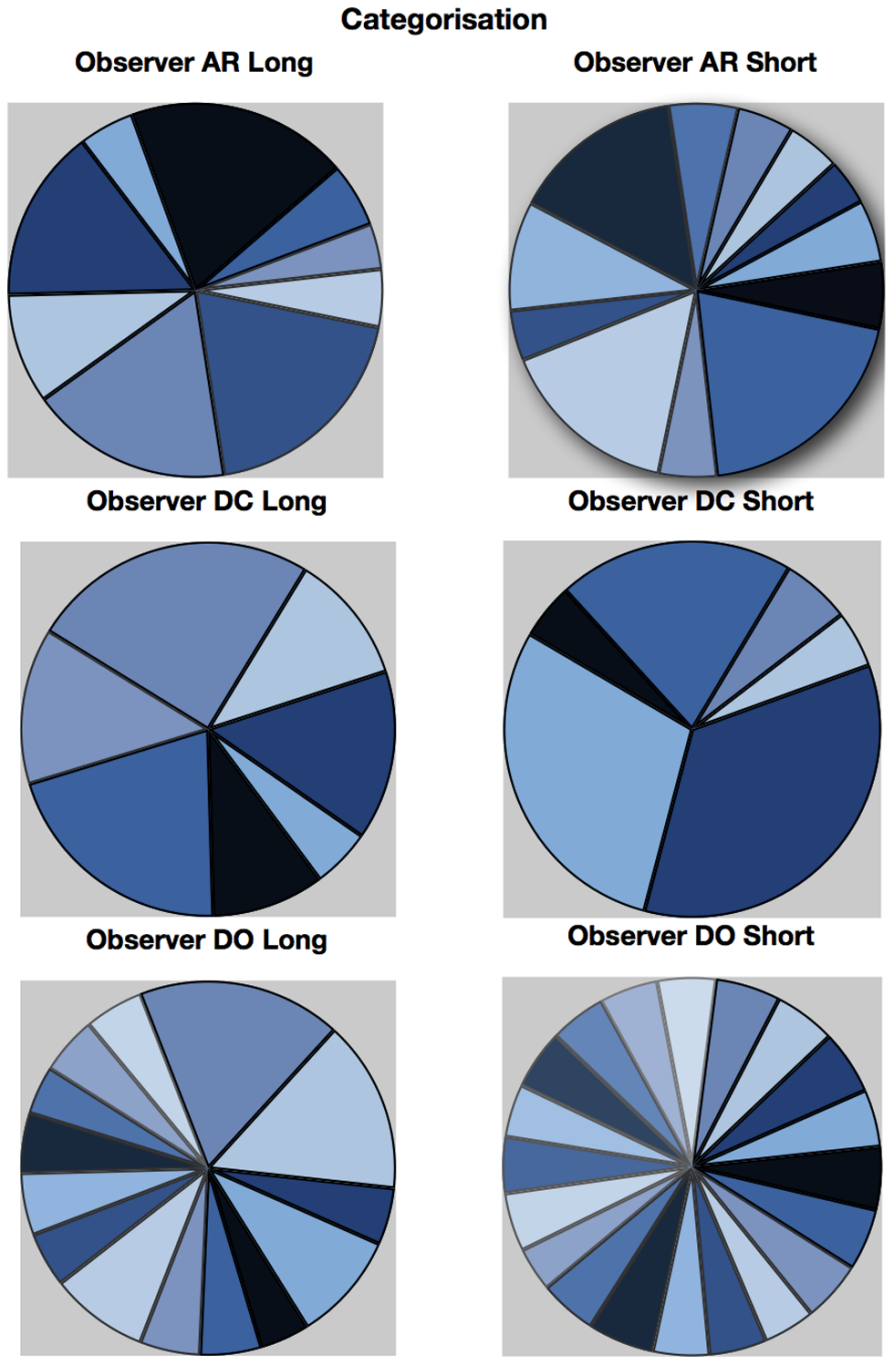
Estimated category boundaries for each observer and duration. See text for details of calculation.

Figure 1 plots the data from the discrimination task in the long (500 ms) condition for three observers. The colour angle of the test stimulus is plotted against the proportion of trials that the observer considered the test and reference stimuli to be physically the same in polar coordinates. Data for the categorisation task in the 500 ms condition is plotted similarly in Figure 2. Figures 3 and 4 plot discrimination and categorisation performance for the same three observers in the short, 50 ms, condition. Figures 5 and 6 summarise (and inevitably approximate) these data as pie charts where the discrimination task performance can be compared to the category boundaries estimated in the categorisation task.

The polar plots are most easily interpreted qualitatively by the degree to which they appear to form a symmetrical and regular petal arrangement as in a flower. The width of the individual petal represents the ‘performance’ in the task, the narrower the petal the greater the sensitivity to the difference between reference and test. Performance is a slightly misleading term as only discrimination ‘performance’ can be thought of in this way, not categorisation. However, in keeping with considering both data sets identically, we retain the term at this stage. The axial symmetry of each petal represents the degree to which the direction around the space affects the performance on either task and the overall regularity of the petal structure indicates how uniform this performance is throughout the colour space. Examining the data in this way reveals that observers identify physical dissimilarity (discrimination) over a much reduced distance around the colour space than they do categorical dissimilarity. It is also the case that categorical dissimilarity is not symmetrical around the reference point nor is it uniform across the colour space or between observers; Figures 5 and 6 emphasise these observations. The computational modeling reported below provides quantitative support for these conclusions.

The effect of stimulus duration appears to be minor for both categorisation and discrimination (bear in mind these stimuli are scaled to their respective contrast detection thresholds) and no systematic variation is obvious for either task. Most critically, for the underlying motivation for varying duration [43], [69], [71], colour categorisation (which we take as a proxy for colour perception) does not simply revert to discrimination at short stimulus durations. There is a suggestion that fewer categories may be clearly identified when the stimulus is on for longer and this may have some relationship to the ability to more clearly and reliably identify a particular colour.

Put most simply, the important point made by Figures 1, 2, 3, 4, 5, and 6 is that these two tasks, discrimination and categorisation, yield different results even though they are performed with exactly the same stimulus set; only the observers' internal decision process changes between the two conditions, not the stimulus input.

A related, and critical, characteristic of the data to notice is that the category data are quite idiosyncratic to each observer whereas the discrimination data are much more similar between observers, as we would expect; categorical judgements are far more subjective than the objective measure of discrimination. Both these characteristics of the data are present in all of the 19 subjects measured to date (publications in preparation), and would be present regardless of the method by which a category or JND boundary were delineated. The discrimination data are generally consistent with other recent work [86], [87]; the categorisation data are entirely novel.

Finally, Table 1 expresses the difference between discrimination and categorisation in statistical terms through the output of a paired-sample t-test comparing percentage of ‘same category’ response in the categorisation task with unbiased percentage probability of ‘same’ response in the discrimination task. All the differences were significant (with a p<0.001) indicating that categorisation and discrimination tasks yielded significantly different data for all observers and conditions.

**Table 1:** Results of a paired 2-tailed T-test comparing discrimination (dis) to categorisation (cat) for each observer (AR, DO, DC) at long and short stimulus durations.

		Paired Differences							
		Mean	Std. Deviation	Std. Error Mean	95% Confidence Interval of the Difference		t	df	Sig. (2-tailed)
					Lower	Upper			
Pair 1	Long duration ARdis - ARcat	−26.93182	16.60946	1.11981	−29.13880	−24.72483	−24.050	219	.000
Pair 2	DOdis - DOcat	−34.50000	27.00900	1.82095	−38.08882	−30.91118	−18.946	219	.000
Pair 3	DCdis - DCcat	−45.26136	32.63215	2.20006	−49.59736	−40.92537	−20.573	219	.000
Pair 1	Short duration ARdis - ARcat	−26.84091	18.15452	1.22398	−29.25319	−24.42863	−21.929	219	0.000
Pair 2	DOdis - DOcat	−20.31818	41.00633	2.76465	−25.76690	−14.86946	−7.349	219	0.000
Pair 3	DCdis - DCcat	−36.73864	22.55548	1.52069	−39.73570	−33.74158	−24.159	219	0.000

All values significant (p<.001).

### JNDs and the Category Boundary Effect

As we have outlined, one of the hallmarks of categorical perception is the increased sensitivity to physical stimulus difference (discrimination) across a category boundary. Quantitatively, discrimination JNDs are relatively evenly distributed throughout the colour plane with a mean degree value for each observer of DC (Long 16.76 ±0.95; Short 16.54±1.43), DO (20.58±1.06; 32.21±1.56) and AR (30.71±1.42; 31.17±1.18) and are symmetrical about the reference (see modeling section). There is the suggestion of an (non-significant) trend toward higher JNDs around the horizontal S-(L+M) axis (90–270 deg) compared to the vertical L-M axis [86], [87] but the distribution of JNDs is far more even than the categorisation data which is entirely subjective and asymmetrical about the reference stimulus. Again, it should be stressed that while the choice of reference stimulus, in terms of its colour, clearly influences the data and the ‘category’ placement in the whole colour plane, comparing the discrimination and categorisation data for the same stimulus set ameliorates this issue: Any category boundary effect, causing enhanced discriminability across boundaries, should be revealed in the discrimination data by asymmetry in the individual curves in the proximity of a boundary. This is not seen in the data either qualitatively or quantitatively and this is examined in more depth in the modeling section. In a parallel study the JNDs were measured directly across and within the boundaries and we found no boundary effect [88].

The primary difference between the categorization and discrimination data can be efficiently summarized as follows: The generalisation gradients (i.e., the proportion of same responses) in the discrimination condition drop off quickly in the discrimination condition. Further, these gradients are relatively symmetric in the discrimination condition. By contrast, the generalisation gradients in the categorisation condition are characterised by marked asymmetry and drop off much slower as one moves further from the reference stimulus (see Figures 1, 2, 3, 4).

## Computational Modeling

To characterize the relationship between categorisation and discrimination in the current study and more directly map categorisation generalisation gradients from the reference stimuli, we applied an exemplar model; the Generalized Context Model (GCM; Nosofsky, 1986). The GCM is perhaps the most well-known model of categorisation; this model assumes that categorisation decisions are made by comparing the to-be-categorized probe item to all previously seen exemplars. In a standard, two-category task, the similarity of the probe item is computed to members of both categories, the similarities are summed within each category and the relative summed similarities are used to determine the probability of responding with each respective category. Importantly, the model provides excellent quantitative fits to not only observed categorisation data but also other tasks that may rely on the same types of similarity computations, such as identification, old-new recognition, and same-different discrimination judgments [53], [54], [58] and inductive reasoning [55]–[57].

In the present task, we assume that for both categorisation and discrimination, similarity is computed only between the probe stimulus and the reference stimulus. This assumption has been used previously to model similarity-based processes underlying same-different judgments with other colour stimuli (Munsell colours varying in hue, saturation and brightness; Cohen & Nosofsky, 2000). In that study, the primary focus was on variation in response time to do specific locations of pairs of stimuli in either dense or isolated regions of the stimulus space. Consequently, Cohen and Nosofsky included additional assumptions about the retrieval of previously seen pairs of items in order to account for the variability in response times on trials in which the two stimuli were the same in both discrimination and categorisation (their Experiment 2). By contrast, in the present study, we are concerned with the proportion of ‘same’ responses in the two tasks of categorisation and discrimination rather than response time. In addition, the stimuli compared in each block are equivalent in ‘density’ between trial blocks; although, as we explain below, the stimulus space as a whole and distances between the stimuli in that space play a central role in explaining the observed data. A further distinction between that study and the present study is that Cohen and Nosofsky examined simultaneously presented stimuli from one particular region of colour space, where we examine the relation between categorisation and discrimination across the entire colour space using sequentially presented stimuli and, between conditions, varying the presentation time of each to-be-compared stimulus (the ‘long’ and ‘short’ conditions).

**Figure 7:**
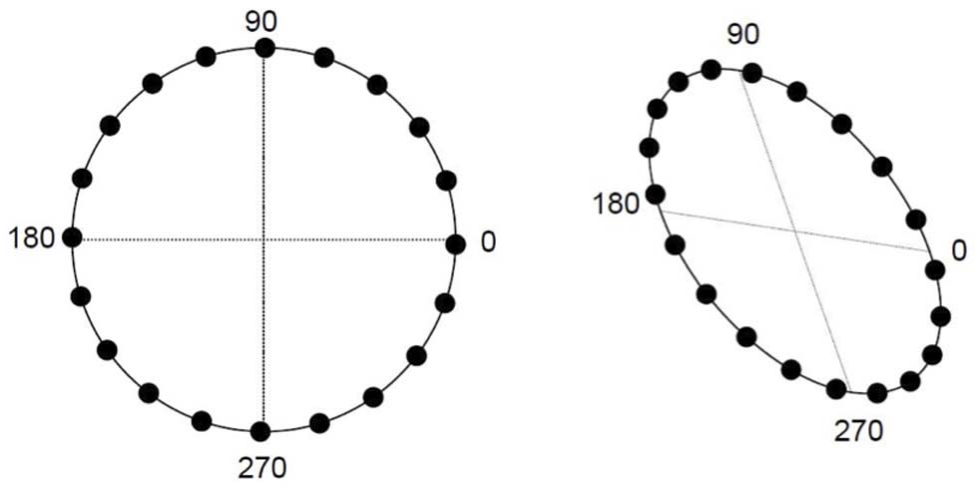
Left panel shows the reference points from each block in cardinal colour space. Right panel shows the reference points from each block in CIE Luv space.

The GCM assumes that stimuli are represented as points on a multidimensional psychological space. Hence, the perceived distances between stimuli can be used to determine their similarity. Although the cardinal colour space captures the opponent-process visual properties of the colour signal when scaled in terms of their subjective detectability, colours represented in cardinal space are not necessarily perceptually uniform in terms of perceived hue. That is, the perceived ‘colour distance’ between two colours is not equivalent in all regions of the cardinal space. Psychological space is usually established through multidimensional scaling of performance to the given stimulus dimensions; however, because the current task requires exhaustive pairwise comparisons, the standard approach is not feasible here. Consequently, in order to appropriately capture the distances between stimuli, we transformed the cardinal colour space to CIE Luv space (cf. [89], [90] and used the transformed values as input to the model (see Figure 7). The u and v dimension of the CIE Luv space do not correspond directly to physiologically or psychologically valid colour dimensions like the opponent axes of the cardinal colour space or the brightness, saturation and hue dimensions of the Munsell colour space, which is often employed in studies of categorisation [50], [51]. Instead, the dimensions of CIE Luv space correspond to transformed axes of CIE XYZ space, which itself is ultimately derived from CIE RGB space. This space is, in turn, constructed from psychophysically-measured colour-matching functions whereby a single monochromatic light is matched by three *primaries* (fixed chromaticity but observer-variable brightness) each predominantly stimulating one of each of the three cone subtypes [91], [92]. The transformations used to derive CIE Luv space result in a more uniform distribution of colours with approximately equivalent perceptual distances in all regions of the colour space [89]

We measured the CIE Luv coordinates, u′ and v′, of the stimuli using a Textronix J18 LumaColourE II Photometer. As only the relative distances between the stimuli are important for modeling the data in the present case, we fit an ellipse to the measured coordinates using a least squares criterion. These coordinates were then standardized in order to ensure that both dimensions had the same range of values. Luminance was not varied; hence, the stimuli are represented solely by the standardized u and v coordinates.

As shown in Figure 7, the coordinates form an ellipse, which implies that in some blocks, colours on one side of the reference stimulus may be perceived to be closer together (i.e., less distant in CIE Luv space) than colours on the other side of the reference stimulus. This provides one hypothesized locus for the asymmetries observed in the categorisation data; namely, stimuli in some regions of the space might be perceived as more similar than stimuli in other regions of the space. Consequently, the probability of responding same may change depending on the location of the colours within the larger colour space. Note that this hypothesis cannot account for the relative symmetry seen in the generalization gradients of the discrimination condition; consequently, the explanation for the difference between categorisation and discrimination must rely on key differences in the parameters assumed to underlie performance in these tasks.

### Model Specification

Formally, the distance between the reference, x_i_, and the test stimulus, x_*j*_ is given by:
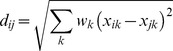
(1)In the present case, the *k* dimensions of *x* are the *u* and *v* coordinates, and the distance is computed by taking the square root of the summed squared differences between the standardized *u* and *v* coordinates weighted by an attention parameter, *w*; the attention parameter is constrained to sum to one across both dimension. The attention parameter adjusts the amount of weight given to each dimension, which may vary due to the elliptical nature of the category space. The similarity between any two stimuli is an exponentially-decreasing function of the distance between the stimuli [93]: 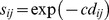
(2)The specificity parameter, *c*, determines how steeply similarity declines with distance (Nosofsky, 1986). High values of c will result in low similarity, even between items which are close together in the stimulus space. Low values are c will result in high similarity even between items which are far apart in the stimulus space. Put another way, for high values of *c*, small differences between stimuli become more salient resulting in lower similarity between stimuli than when c takes on a lower value.

The probability of responding that a probe item either belongs to the same category as the reference stimulus or that a probe item is the same item as a reference stimulus is given by:
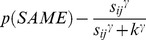
(3)where *k* and *γ* are free parameters. The parameter, *k*, is a criterion used to transform similarity to the probability of responding same. The response scaling parameter, *γ*, allows responding to vary according to the ratio of *s_ij_* and *k*, when *γ* is near one, to deterministic responding when *γ* is greater than one.

In summary, the base version of the model has four free parameters, *w*, *c*, *k*, and *γ*. In typical applications of the GCM to integral-dimensioned stimuli [50], [53], the attention parameter, *w*, is unlikely to play a crucial role; however, because of the elliptical nature of space, across different blocks, the reference point will differ to the comparison stimulus along either or both of the dimensions. Consequently, attention is likely to vary across blocks reflecting the specific location of stimuli within the elliptical space. Categorisation and discrimination are likely to differ on the value of the specificity parameter, *c*, needed to predict the behaviour observed in both tasks with higher values predicted in discrimination than in categorisation. It is also possible that different specificity parameters might be required in different parts of the stimulus space. For example, in some regions of the space, for some observers, especially near the major axis of the ellipse, discrimination performance remains largely symmetrical but large asymmetries are apparent in the categorisation data; the categorisation asymmetries may depend on higher values of the specificity parameter in those regions of the stimulus space. Finally, the criterion and response scaling parameters may vary by task and by block. More specifically, the response scaling parameter is likely to differ between categorisation and discrimination with a lower criterion expected in the former task relative to the latter (a similar difference is found between categorisation and old-new recognition, see e.g., [54]. Consequently, we fit a number of different versions of the model which constrained the model parameters in different ways (i.e., either across blocks or tasks or both). By comparing the fits of the constrained models to an unconstrained model in which all parameters are free to vary across tasks and blocks and to the base version of the model in which all parameters are fixed across task and block, we can reveal which parameters best account for behaviour in both tasks.

### Model fitting procedure

Because the data from each observer are binomially distributed, we used the log of the binomial likelihood to optimize the parameters for the fits to each observer's data: 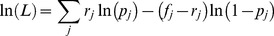
(4)where *p_i_* is the model's predicted probability responding same for item *j*, *r_j_* is the observed number of same responses for item *j*, and *f_j_* is the frequency of item *j* (i.e., the number of times item *j* was presented). Parameters were optimized separately for each observer in each condition using a Hooke and Jeeves algorithm to minimize the negative log likelihood function from a large number of different starting points [94].

We fit a version of the model in which all parameters were allowed to vary by task and by block. This resulted in a very flexible model (hereafter, the *fully flexible* model) with 160 free parameters fit to 440 data points (20 blocks of 11 stimuli in both the categorisation and discrimination tasks). The log-likelihood values of the model were converted to Bayesian information criteria (BIC; [95]) by adding a penalty term which is a function of the number of free parameters in the model, *n_p_*, and the number of data observations being fit, *M*: 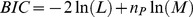
(5)We then systematically constrained parameters across tasks or blocks or both, computed the log-likelihoods for the constrained models and compared the model fits using BIC. The model that yields the smallest BIC is preferred as it balances the fit to the data against complexity due to excess parameters. Any constrained model can never have a maximum likelihood better than the fully flexible model; however, reduction in the number of free parameters results in a lower penalty effectively improving the goodness of the model fit to the data. This approach does not favor any constraint *a priori* but rather allows us to compared different constrained versions of the model and focus on the model which best captures the data without excessive parameters.

### Modeling results

In the fully flexible model, the exponent, γ, in Equation 3 took on a large value across tasks and blocks. Since variation in this parameter has little effect when the parameter is large, we fixed the parameter across the remaining model fits; as expected, the log-likelihoods were unchanged by this constraint (see Table 2). In addition to the fully flexible and fully constrained comparison models, the modeling results indicated that two additional models were particularly relevant for explaining the current results. As shown in Table 2, observer AR's data in the long and short duration conditions and observer DC's data in the short duration condition were best fit by a version of the model in which the attention weight, *w*, and the specificity parameter, *c*, varied by block and the criterion parameter, *k*, was varied across both blocks and tasks (i.e., categorisation and discrimination). This model is hereafter referred to as the *flexible criterion* model. By contrast, observer DC's data in the long duration condition and observed DO's data in the short duration condition were best fit by a version of the model in which attention, *w*, varied by block, specificity, *c*, varied by block and by task and the criterion parameter, k, was fixed across task; hereafter, this model is referred to as the *flexible specificity* model. Observed DO's long duration results were best fit by the fully flexible model.

**Table 2:** Model fits (-lnL and BIC) for the best fitting model for each observer and duration.

						Long Duration					
	Parameters					AR		DC		DO	
Model	c	k	w	λ	M	-lnL	BIC	-lnL	BIC	-lnL	BIC
Fully Flexible	TxB	TxB	TxB	TxB	160	7307	15588	5819	12612	5763	12499
Fixed λ	TxB	TxB	TxB	F	121	7307	15350	5819	12375	***5763***	***12262***
Flexible Specificity	TxB	T	B	F	63	7426	15236	***5944***	***12272***	6007	12398
Flexible Criterion	B	TxB	B	F	81	***7368***	***15228***	5909	12311	5911	12314
Fully Constrained	F	F	F	F	4	9260	18544	10531	21086	10095	20214

						Short Duration					
	Parameters					AR		DC		DO	
Model	c	k	w	λ	M	-lnL	BIC	-lnL	BIC	-lnL	BIC
Fully Flexible	TxB	TxB	TxB	TxB	160	7502	15978	6630	14233	6284	13541
Fixed λ	TxB	TxB	TxB	F	121	7502	15741	6630	13996	6284	13304
Flexible Specificity	TxB	T	B	F	63	7666	15714	6761	13905	***6332***	***13047***
Flexible Criterion	B	TxB	B	F	81	***7583***	***15658***	***6696***	***13884***	6346	13184
Fully Constrained	F	F	F	F	4	10422	20868	10706	21436	9887	19798

Notes: *c* - specificity parameter, *k* -criterion parmeter, *w* - attention weight and *λ*, response exponent. TxB - Task x Block, T - Task, B - Block, F - Fixed across task and block. M – number of parameters, -lnL - Negative log-likelihood, BIC - Bayesian Information Criterion.

The best fitting model (i.e., lowest BIC) is shown in bold.

**Figure 8:**
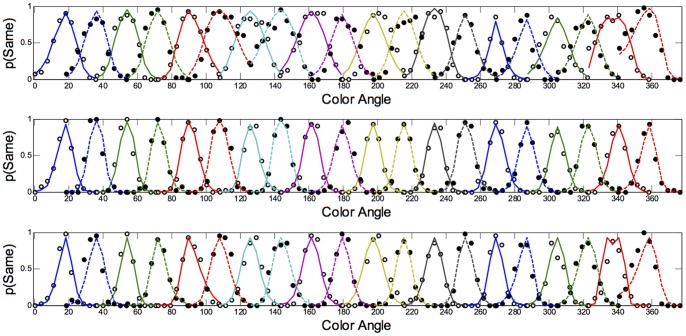
Discrimination Long Duration (500 ms): Predictions of the model to each of the blocks for Observers AR (top panel), DC (middle panel) and DO (bottom panel). Data are shown as dots; the model predictions are shown as lines fit to the data. Color and style of lines and dots are alternated for clarity.

**Figure 9:**
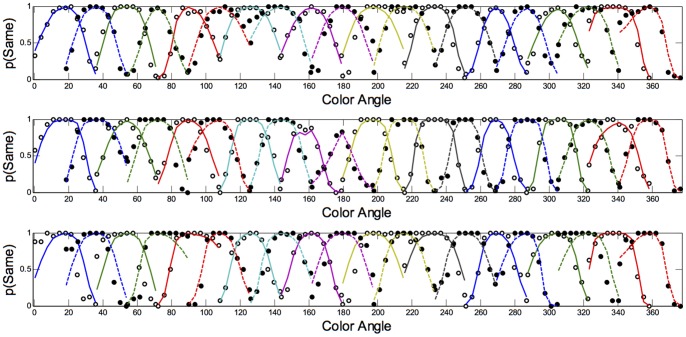
Categorization Long Duration (500 ms): Predictions of the model to each of the blocks for Observers AR (top panel), DC (middle panel) and DO (bottom panel). Data are shown as dots; the model predictions are shown as lines fit to the data. Color and style of lines and dots are alternated for clarity.

**Figure 10:**
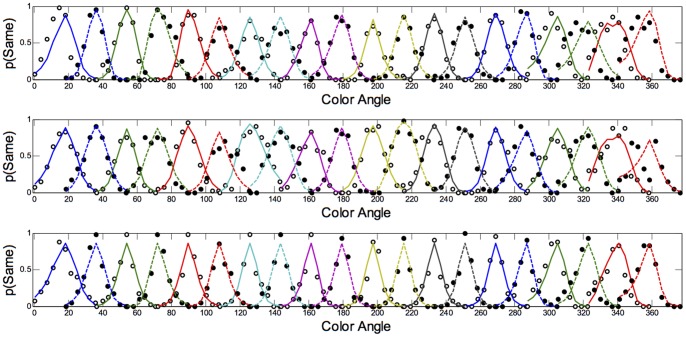
Discrimination Short Duration (50 ms): Predictions of the model to each of the blocks for Observers AR (top panel), DC (middle panel) and DO (bottom panel). Data are shown as dots; the model predictions are shown as lines fit to the data. Color and style of lines and dots are alternated for clarity.

**Figure 11:**
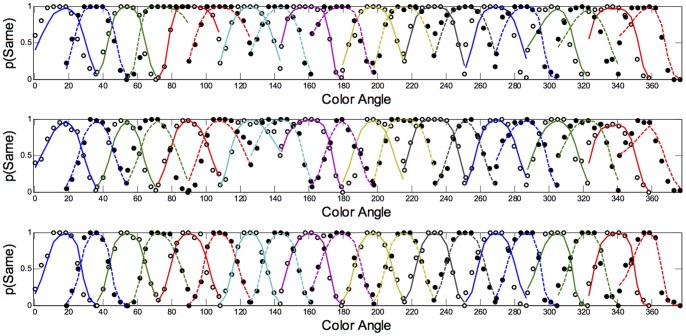
Categorization Short Duration (50 ms): Predictions of the model to each of the blocks for Observers AR (top panel), DC (middle panel) and DO (bottom panel). Data are shown as dots; the model predictions are shown as lines fit to the data. Color and style of lines and dots are alternated for clarity.

The fits of the model with the lowest BIC to each observer's discrimination and categorisation data are shown in Figures 8, 9, 10, and 11. As is evident from the figures, the model accurately captures the generalization gradients across the discrimination and categorisation data. In support of this claim, we computed the root mean squared deviations (RMSD) between the model predictions and the data for each block. The average RMSD for each observer in each condition are shown in Table 3. The order of RMSD roughly follows the amount of asymmetry present in the generalization curves with lower RMSD's (i.e., better fits) for more symmetrical curves. Inspection of the model fits within each block revealed a small proportion of the blocks (.07) had RMSDs larger than.15. These poor fits were generally in blocks in which the participant showed extensive asymmetry, but the model only predicted mild asymmetry or asymmetry in the wrong direction. Consequently, although the model can predict asymmetries based on smaller distances between the stimuli on one side of the reference point than the other, the model does not adequately handle all of the asymmetry and in some cases, predicts qualitatively different asymmetry than what is observed. Although this is clearly a failing of the model, it provides a compelling clue about the types of additional or alternative mechanisms that may be influencing the categorisation behaviour; we return to these mechanisms below.

**Table 3:** Root mean-squared deviation (RMSD) averaged across blocks for each observer and duration.

	Long Duration			Short Duration		
	AR	DC	DO	AR	DC	DO
Categorization	0.09 (0.05)	0.13 (0.09)	0.08 (0.04)	0.09 (0.05)	0.10 (0.05)	0.08 (0.04)
Discrimination	0.10 (0.04)	0.09 (0.04)	0.07 (0.03)	0.10 (0.04)	0.10 (0.06)	0.07 (0.03)

### Model parameters

#### Attention parameter

**Figure 12:**
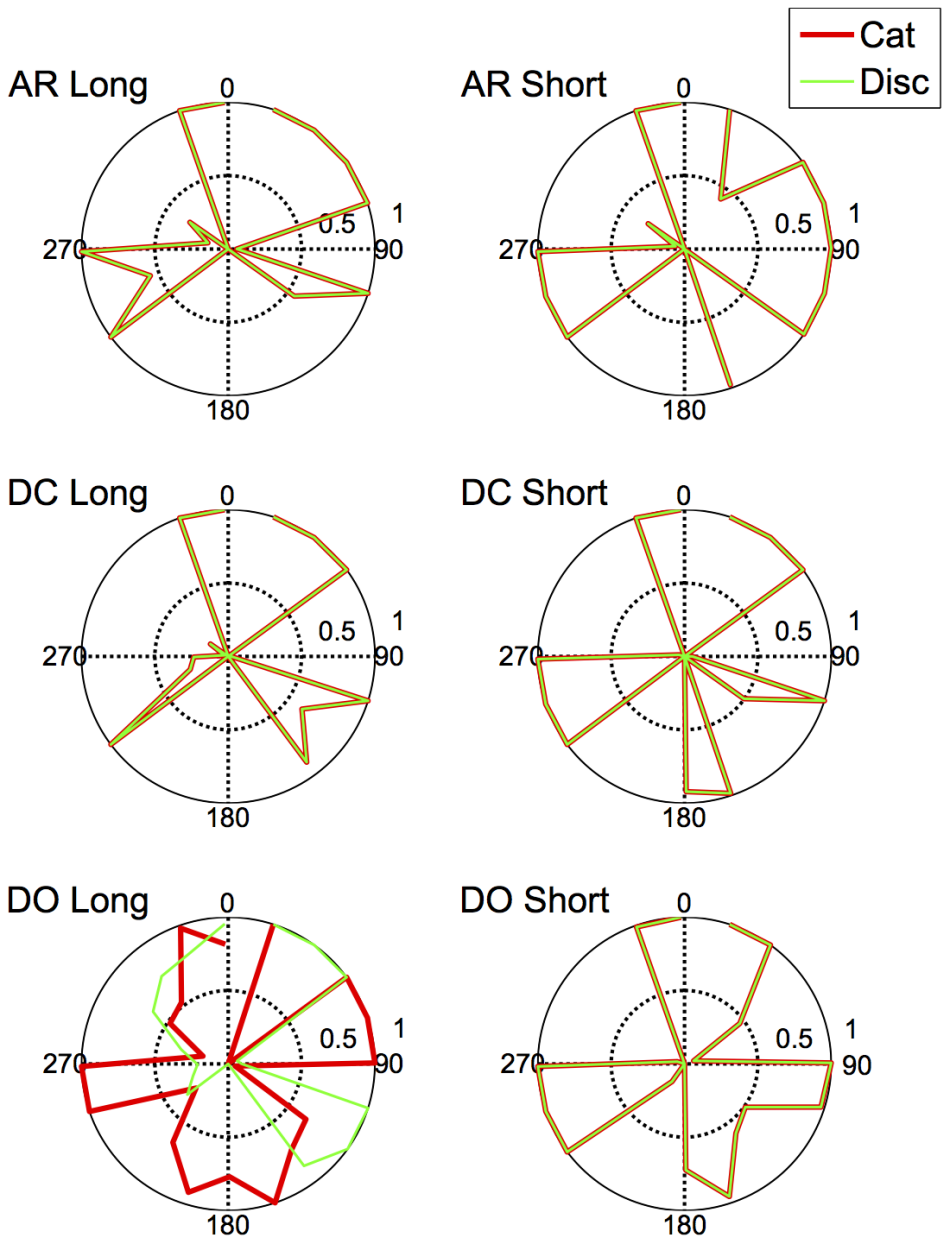
Attention parameter, *w*, from the best fitting model (see text for details) plotted at each reference point for the categorization condition (red) and discrimination condition (green). Left hand panels show the long duration condition; right panels show the short duration condition.

The attention parameters for each observer and each condition are shown in Figure 12. The attention parameters are highly consistent across observers and conditions with attention near unity (i.e., complete attention to the u dimension) particularly for blocks that have reference points between 0 and 90 degrees and near 270 degrees. Consistent near zero attention weights (i.e., complete attention to the v dimension) is found for blocks that have reference points between 180 and 270 and between 270 and 360 degrees. A small number of other blocks show varied attention weights between observers and conditions; however, the consistency across a large number of blocks suggests that the attention weight is primarily influenced by the position of colours within the elliptical colour space. This is logical as it makes little sense for the model to heavily weight dimensions on which the colours within a block show only small variation.

#### Specificity

**Figure 13:**
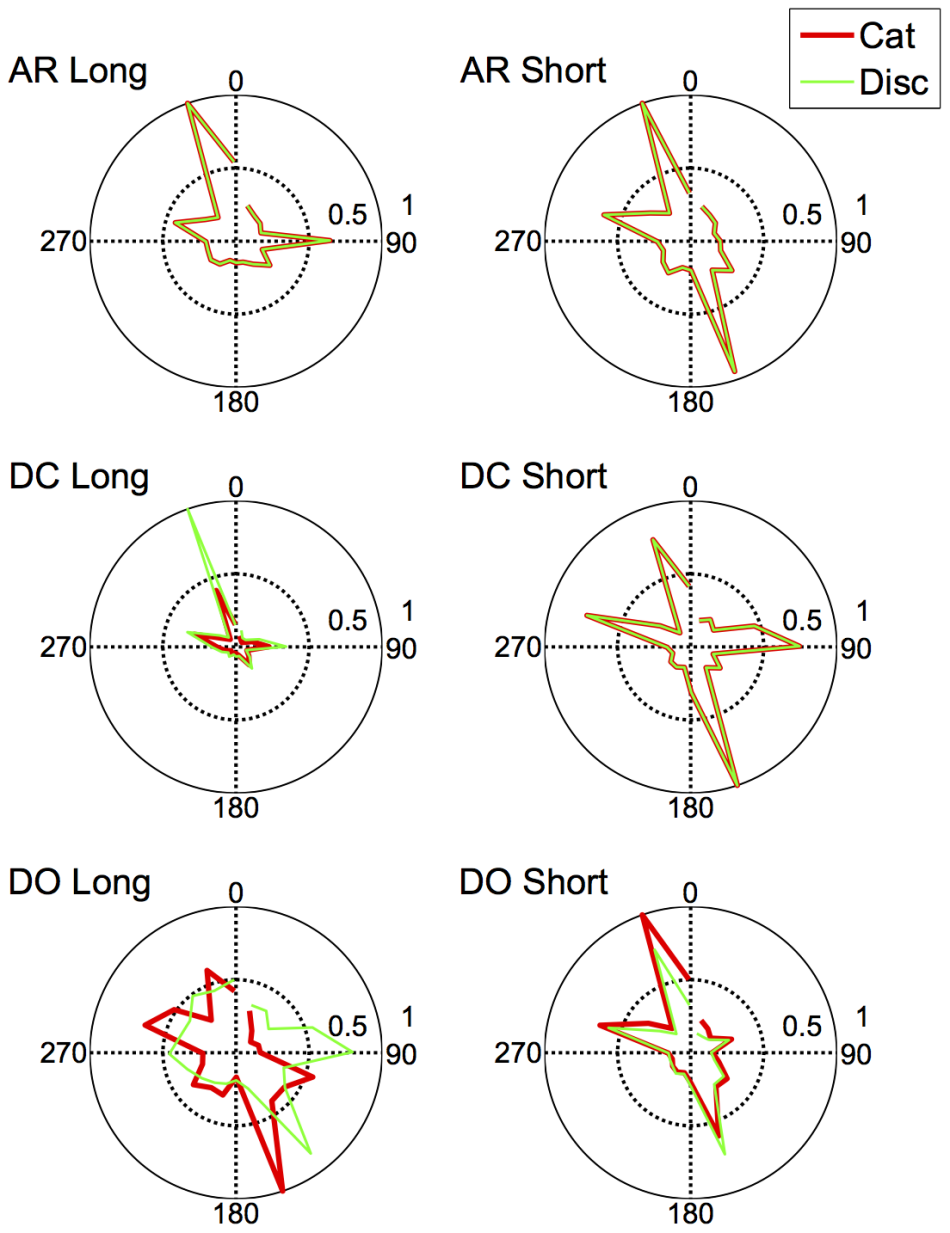
Specificity parameter, *c*, from the best fitting model (see text for details) plotted at each reference point for the categorization condition (red) and discrimination condition (green). Left hand panels show the long duration condition; right panels show the short duration condition. The specificity parameters were standardized to have a maximum value of 1 by dividing by the maximum estimated specificity (i.e., *c_max_* = [45.31, 57.38, 15.86] for observers AR, DC and DO in the long duration condition and *c_max_* = [34.25, 407.2, 23.59] for observers AR, DC and DO in the short duration condition).

The specificity parameter is also consistent across observers and durations with all observers showing distinct peaks of various magnitude in the value of the specificity parameter for some blocks, specifically those blocks with reference colours at 90, 160, 286 and 340 degrees (see Figure 13). The specificity parameter did not vary dramatically between the categorisation and discrimination conditions, with three of the observer/duration conditions better fit by a model in which specificity did not vary by task. For the other three observer/duration conditions, the differences between the tasks are small. The specificity parameter adjusts the steepness in the exponential similarity function; consequently, high values of this parameter in specific locations may represent areas of the space in which the observer is most sensitive to small changes in the stimulus. It is unclear why the specificity parameter peaks at the locations shown in Figure 13; however, we offer some possibilities in the General Discussion.

#### Criterion

**Figure 14:**
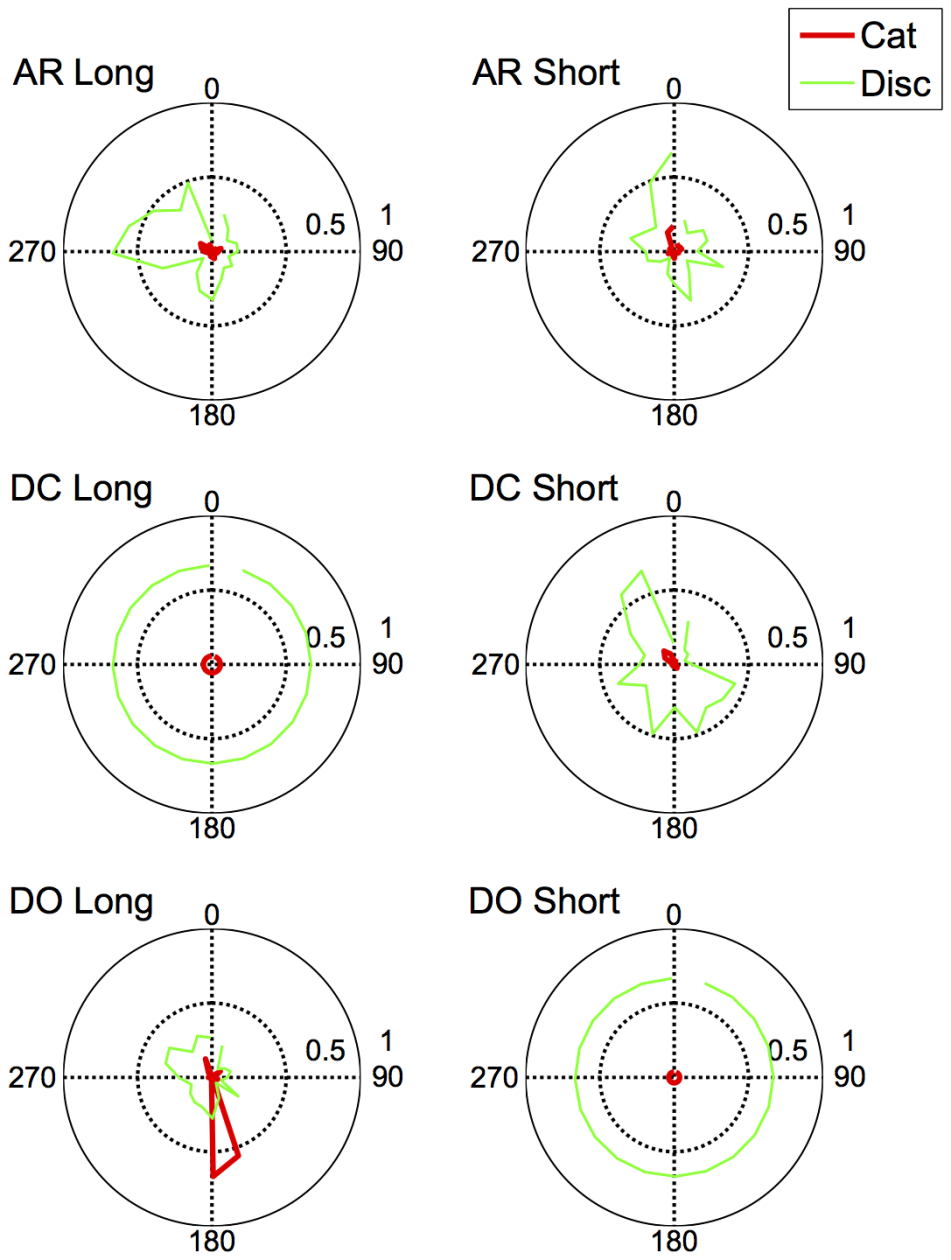
Exponentiated criterion parameter, *k^γ^*, from the best fitting model (see text for details) plotted at each reference point for the categorization condition (red) and discrimination condition (green). Left hand panels show the long duration condition; right panels show the short duration condition. The response scaling parameter, γ, parameter for each observer was as follows: Long duration condition = [1.85, 5.32, 6.02] for observers AR, DC, and DO, respectively. Short duration condition = [2.10, 0.25, 5.17] for observers AR, DC, and DO, respectively. The exponentiated criterion parameter was scaled to be comparable to the other parameters by dividing by the 1.5 times the maximum estimated exponentiated criterion (i.e., *k^γ^_max_* = [0.40, 0.29, 0.16] for observers AR, DC, and DO, respectively, in the long duration condition and *k^γ^_max_* = [0.40, 0.29, 0.18] for observers AR, DC, and DO, respectively, in the short duration condition).

As shown in Equation 3, the criterion parameter in the denominator is exponentiated. Since the exponent is constant across blocks and tasks, we plot the exponentiated criterion in Figure 14. Consistent with expectation, the criterion in the discrimination conditions is consistently higher than in the categorisation conditions (with the exception of two blocks for observer DO's long duration data). This is consistent with the idea that a “same” decision in discrimination requires more confirmatory evidence than in categorisation (cf. [54]).

In summary, there is remarkable consistency in the parameters across and within observers and across tasks. Consistency in the attention parameter is largely the result of constraints imposed by the elliptical nature of the colour space. Consistency in the specificity parameter may also be due to specific aspects of the category space; however, the peaks in the specificity parameter do not appear to be tied directly to observed asymmetry in the data, nor to specific areas of the elliptical category space in which the colours are compressed. The criterion required for discrimination is consistently higher in discrimination than in categorisation.

Despite the having parameters which vary across blocks and tasks, the model makes qualitatively inaccurate predictions in some of the blocks predicting asymmetries which are in the opposite direction to that actually observed. This occurs only in the categorisation condition; by contrast, the high discrimination criterion allows the model to accurately predict the symmetrical generalization gradients for the discrimination data. It is important to note that the model can only predict asymmetry when stimuli on one side of the reference point are closer together than stimuli on the other side. Further, the model can only predict asymmetry in the direction of the compressed stimuli (i.e., the model predicts that the probability of responding same should be higher in the direction of the compressed stimuli than in the opposite direction). To characterize the degree of asymmetry which is not well-predicted by the model, we created a measure of stimulus compression by finding the distance between the two stimuli furthest to the left of the reference stimulus (*d_1,2_*) and the two stimuli furthest to the right of the reference stimulus (*d_10, 11_*) using Equation 1. We then identified blocks with compression to the left (*d_1,2_<d_10,11_*) or to the right (*d_1,2_>d_10,11_*). We also computed a measure of asymmetry by finding blocks in which different in the probability of responding “same category” between items 1 and 11, *x_1_* and *x_11_*, was greater than or less than some asymmetry threshold (i.e., how much difference is necessary before classifying the data as asymmetrical). We then computed the proportion of blocks in which the compression and the asymmetry of responding same category were in the same direction. These proportions vary according to the size of the asymmetry threshold (i.e., because the number of blocks showing asymmetry decreases with the asymmetry threshold); hence, we used a range of thresholds from .2 to .4. Averaged across the threshold range, observer DC had the most asymmetrical blocks with 13 and 12 in the long and short duration conditions, respectively, observer AR had 8 and 9 asymmetrical blocks in the long and short duration condition, respectively, and observer DO had an average of 4 and 1 asymmetrical blocks in the long and short duration conditions, respectively.

**Figure 15:**
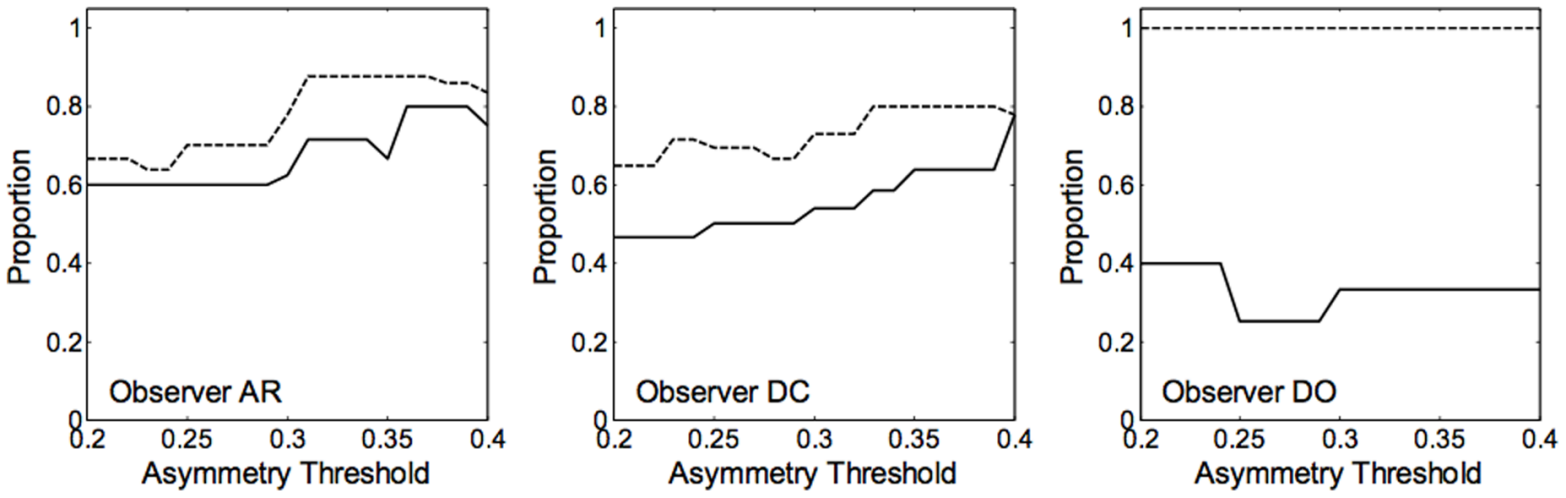
Proportion of blocks in which the observed asymmetry is in the same direction as compression in the stimulus space in the categorization condition for each observer. The dotted line shows the short duration condition; the solid line shows the long duration condition.

As shown in Figure 15, for observers AR and DC, compression in the stimulus space is in the same direction as the asymmetry in over half of the asymmetrical blocks and completely in the same direction for the short duration condition for observer DO. In addition, these proportions are always higher in the short duration condition than in the long duration condition across all observers implying that extra observation times in the long duration condition allows for some aspects of the colour signal to influence processing in a manner which results in asymmetries which do not accord with the compression in the category space.

### Modeling Summary

What then does the modeling tell us about behaviour?

1. The model fully characterizes the primary differences between categorisation and discrimination as a difference in criterion, with a lower criterion required for making a ‘same’ response in categorisation than in discrimination.
2. Asymmetries in the categorisation data are primarily explained by the elliptical nature of the stimulus space when plotted on a particular (CIE Luv) set of axes; that is, due to the shape of the colour space, in some blocks stimuli on one side of the reference are spaced further apart than stimuli on the other side of the space resulting in a lower similarity and the reference and a sharper drop-off in the proportion of same response for those stimuli.
3. For blocks in which the data (including the asymmetry in the categorisation data) are well-predicted by the model, the implication is that the responses are based solely on comparison between the target and reference stimuli.
4. There remain some asymmetries which are not well-predicted by the model, and this is true in the long duration more than the short duration condition, suggesting that whatever is driving the asymmetry is a consequence of having extra time to process the stimulus. There are at least two possible explanations for the mispredicted asymmetry. First, our assumptions about the spacings of the stimuli in CIE Luv space may be incorrect. Individuals most likely vary in the perceived distances between the stimuli; consequently, in some blocks, the CIE Luv space, which is a derived space based on averaged data may not be appropriate for some observers. A second possibility is that there are regions of the stimulus space for which observers are influenced by information other than that contained in the colour stimulus. For example, if the reference and the target colour belong to different categories specified by a unique hue boundary, then asymmetries might result simply from the stimuli within a block crossing over that boundary (we address this further below).

## General Discussion

The work presented in the paper has relevance both to the study of colour vision and to the examination of categorisation of stimuli in general.

### Colour

What we have shown is that observers are able consistently to perform a categorisation task on a stimulus set largely used for simple tasks of colour perception (detection and discrimination). Not only do the data reveal that there is some measurable intermediate stage of colour sensation prior to learned colour-naming but that this psychophysical ability does not affect discrimination in any way; there is no evidence for a category-boundary effect in the data [19], [88]. This suggests that the oft-cited categorical nature of colour perception needs to be clearly qualified as being a property of a named and learned colour stimulus-set, which in turn makes it a quite restricted and far less interesting property from the perspective of human colour vision and is perhaps far more founded in language and communication, around which there is significant debate [44], [65], [96]–[103]. When considered in the context of the ‘problem’ faced by the visual system, or any sensory system [104], this observation is less contentious if the purpose of categorical perception is to optimise the system performance by effectively enhancing sensitivity across a boundary at the expense of reducing sensitivity between the boundaries [19]. Such an approach makes good sense for stimuli such as phonemes, when judgement of the stimulus characteristics must be fast and effective in order to understand speech, but makes no logical sense for the fine graded perception of colour (and brightness) in a complex scene. In this paper we offer an alternative explanation of the categorisation and discrimination data which assumes that same/different decisions in both tasks are based on a common similarity-based mechanism.

The relatively uniform nature of the discrimination space compared to the asymmetrical and quite idiosyncratic form of the free-categorical space suggests that the observers are clearly performing different tasks even though the stimulus set remains unchanged for each task. We suggest that the free-categorisation task examined here may be the simplest form of colour sensation, and provides some intermediate psychophysical measure of colour perception between discrimination and colour-naming, and we speculate that its neural analogue may be the population response of colour selective neurones seen in V1 [105], [106], a suggestion made previously in the context of chromatic motion discrimination [107].

As outlined in the context of the motivation for this study, the perception of colour, as opposed to the detection and discrimination of different coloured stimuli, has generally, although not exclusively, been examined using stimuli specifically constructed around a perceptual colour space, initiated from an artistic perspective by Albert Munsell [108]. There are extensive data on the behavioural properties of stimuli such as these both in isolation and in the context of different environments and illuminants (see [14] for a thorough recent review). The difficulty has been, to date, to link these perceptual data to the detection and discrimination data, at least in part due to between-task differences in the stimuli and conditions [8], [9], [12]. From a task perspective the obvious overlap between the two approaches has been the Just Noticeable Difference (JND) which forms the scalar of the perceptual space, and is the result of any discrimination task in cone or cardinal space. Here we have taken this overlap one stage further with the free-categorisation task.

Other recent approaches to this issue of bridging the gap between the two streams of research have included naming colours represented in a cone-contrast space [33], [42] and mapping unique hues [34], modelling the response of LGN neurones to Munsell colour chips [5], [6], and examining the effect of prolonged adaptation to the perception of Unique Yellow [109]. Several studies have looked at the ability of observers to classify cone modulating stimuli in terms of how much of two opponent colours (eg Red-Green, Blue-Yellow or Black-White) they are thought to contain [39], [40], [42], [49]. Observers are able to perform the task, to perceptually extract a given hue, even though the coloured stimulus is itself perceptually mixed (Malkoc & Kingdom, 2012). In each of these studies the observers were required to use their internal representation of the named hue to perform the task. The current study extends these studies in two ways; we allow and encourage the observers to define their own category and discourage the use of remembered named colours, and we measure the difference between discrimination and categorisation around the circumference of a colour ellipse rather than effectively across the middle, as is the case with opponent hues. While we accept that observers are free to use named colours if they wish, we do not consider this to be the case. In a subsequent study when observers were given colour names for previously unnamed stimuli; the rate at which they learned a new category increased significantly although the category properties themselves remained the same (Unpublished data; manuscript in preparation). In terms of the suggestions made by the modelling, it also seems far more intuitive for the observers to use a more general definition of category such as of degree of similarity between two perceptually mixed colours, than try to identify a particular characteristic hue within a mixed stimulus, upon which to make their judgement. The large stimulus array and presentation structure also suggests this might be the most straightforward approach to the task; future work is aimed at disentangling this issue.

In summary, each of the studies cited above, as well as the current study, has contributed to the process of mapping the development of the colour ‘signal’ in the system and all largely conclude that the perception of colour is a multi-dimensional property which exhibits characteristics of every stage of the process in its measured properties, whichever behavioural task is chosen. The current data are congruent with this view in that the task of categorisation is shown to be a property of cardinal stimuli and operates as a ‘stripped down’ version of the behavioural measure, showing no category boundary effect, when the current stimuli and conditions are used.

### Categorisation

The data revealed two primary differences between the discrimination and categorisation data: Firstly, the curves within each block were much narrower in the discrimination task than in the categorisation task. This reflects highly accurate performance in the discrimination task (i.e., responding ‘same’ only when the test colour was very similar to the reference colour). In the categorisation task, the curves were wider indicating a tendency to endorse test colours which were further from the reference colour (in comparison to discrimination) as belonging to the same category. This result is qualified by, secondly, a marked asymmetry in the curves for the categorisation condition but not the discrimination condition.

The computational modeling revealed a close correspondence in the parameter estimates across observers and within observers across task durations. This is the first examination of how these model parameters change across the entire color space; consequently, the parameter estimates reported here offer a key benchmark for future research. Namely, the discrimination criterion is higher than the categorization criterion explaining the steep discrimination generalization gradients and the relatively shallow categorization generalization gradients. The attention parameter varies in specific locations of the stimulus space influence primarily by the elliptical nature of CIE Luv space. We note that the specificity parameter is increased in blocks which roughly fall between the unique hue locations as measured by Malkoc and Kingdom (2012). Consequently, increased specificity might indicate the location of a boundary between two unique hue regions, though these regions are not equivalently-sized. For instance, the locations of unique red and unique yellow are near 0 (or 360) degrees and 300 degrees, respectively, but specificity is peaked near 340 degrees [35] By contrast, the peak between unique red and unique blue (at 125 degrees) is around 90 degrees. This could indicate a bias in how the broadband color stimuli are allocated to the unique hue categories (e.g., it might be reasonable for a “red” stimulus to contain a substantial amount of blue but it is more likely to be called “yellow” if it contains even a small amount of yellow). This would imply the unique (or best example) colors do not fall in the center of their respective categories. We leave this as a direction for future research which might address this question by adequately measuring both the distances between the stimuli in a uniform colour space and the locations of unique hues in relation to the reference and target colour locations.

## Conclusions

From the stimulus-driven perspective of colour, the data show there is some intermediate stage of colour sensation prior to, and possibly functionally independent of, colour-naming and that this psychophysical ability does not affect discrimination in any way.

From the task-driven perspective of categorisation and its relationship to other forms of stimulus recognition and identification, the data provide a clear quantitative link to the simpler task of discrimination and give a more coherent behavioural analogue of the development of a neural representation of a stimulus. While discrimination and categorisation in the same stimulus set are statistically different decision-processes, they can be delineated from each other simply on the basis of prior knowledge of the stimulus set; in this case the sensory quality of colour.

Strongly influenced by Hering, we are encouraged that this approach contributes toward a unifying framework for colour perception, which in turn gives an unusually coherent exemplar of the nature of stimulus detection, discrimination, categorisation and identification.

## References

[1] Hering E (1964) Outlines of a theory of the light sense. Hurvich LM, Jameson D, translator. Cambridge, MA: Harvard Univeristy Press.

[2] Hering E (1878) Zur Lehre vom Lichtsinn. Wein: Gerald und Sohne.

[3] Helmholtz Hv (1925) Helmholtz's treatise on physiological optics. Southall JPC, translator. Washington DC, USA: The Optical Society of America.

[4] Helmholtz Hv (1896) Handbuch der Physiologischen Optik 2nd ed.). Hamburg: Voss.

[5] Romney AK, D'Andrade RGD, Indow T (2005) The distribution of response spectra in the lateral geniculate nucleus compared with reflectance spectra of Munsell color chips. Proceedings of the National Academy of Sciences of the USA 102: 9720–9725. 15976023 10.1073/pnas.0503887102 PMC1172267

[6] Romney AK, D'Andrade RGD (2005) Modeling lateral geniculate nucleus cell response spectra and Munsell reflectance spectra with cone sensitivity curves. Proceedings of the National Academy of Sciences of the USA 102: 16512–16517. 16263925 10.1073/pnas.0508172102 PMC1283460

[7] Wandell BA, Chichilnisky EJ (2012) Squaring cortex with color. Nat Neurosci 15: 809–810. 22627792 10.1038/nn.3124

[8] Valberg A (2001) Unique Hues: an old problem for a new generation. Vision Research 41: 1645–1657. 11348647 10.1016/s0042-6989(01)00041-4

[9] Eskew RT Jr (2009) Higher order color mechanisms: a critical review. Vision Research 49: 2686–2704. 19616020 10.1016/j.visres.2009.07.005

[10] Gegenfurtner KR, Kiper DC (2003) Color vision. Annu Rev Neurosci 26: 181–206. 12574494 10.1146/annurev.neuro.26.041002.131116

[11] Conway BR (2009) Color vision, cones, and color-coding in the cortex. Neuroscientist 15: 274–290. 19436076 10.1177/1073858408331369

[12] Shevell SK, Kingdom FA (2008) Color in complex scenes. Annu Rev Psychol 59: 143–166. 18154500 10.1146/annurev.psych.59.103006.093619

[13] Steels L, Belpaeme T (2005) Coordinating perceptually grounded categories through language: A case study for colour. Behavioural Brain Sciences 28: 469–529. 10.1017/S0140525X05000087 16209771

[14] Foster DH (2011) Color constancy. Vision Res 51: 674–700. 20849875 10.1016/j.visres.2010.09.006

[15] Seaborn M, Hepplewhite L, Stonham J (2005) Fuzzy colour category map for the measurement of colour similarity and dissimilarity. Pattern Recognition 38: 165–177.

[16] Saunders BAC, van Brakel J (1997) Are there non trivial constraints on colour categorisation. Behavioural Brain Sciences 20: 167–228. 10096997

[17] Ashby FG, Lee WW (1991) Predicting similarity and categorization from identification. J Exp Psychol Gen 120: 150–172. 1830609 10.1037//0096-3445.120.2.150

[18] Maddox WT, Ashby FG (1996) Perceptual separability, decisional separability, and the identification-speeded classification relationship. J Exp Psychol Hum Percept Perform 22: 795–817. 8756953 10.1037//0096-1523.22.4.795

[19] Harnad S (1987) Categorical Perception. Cambridge, UK.: Cambridge University Press.

[20] Nosofsky RM (1986) Attention, similarity, and the identification-categorization relationship. J Exp Psychol Gen 115: 39–61. 2937873 10.1037//0096-3445.115.1.39

[21] Goldstone RL, Hendrickson AT (2010) Categorical Perception. Interdisciplinary Reviews: Cognitive Science 1: 65–78. 10.1002/wcs.26 26272840

[22] Krauskopf J, Williams DR, Heeley DW (1982) Cardinal directions of color space. Vision Research 22: 1123–1131. 7147723 10.1016/0042-6989(82)90077-3

[23] Krauskopf J, Gegenfurtner K (1992) Color discrimination and adaptation. Vision Res 32: 2165–2175. 1304093 10.1016/0042-6989(92)90077-v

[24] Krauskopf J, Srebro R (1965) Spectral sensitivity of color mechanisms: Derivation from fluctuation of color appearance near threshold. Science 150: 1477–1479. 5853939 10.1126/science.150.3702.1477

[25] Krauskopf J, Williams DR, Madler MB, Brown AM (1986) Higher order color mechanisms. Vision Research 26: 23–32. 3716212 10.1016/0042-6989(86)90068-4

[26] Krauskopf J (1999) Higher Order Color Mechanisms. In: R GK, Sharpe LT, editors. Color Vision, From Genes to Perception. Cambridge, UK: Cambridge Univesity Press. pp. 149–164.

[27] Hurvich LM, Jameson D (1957) An opponent-process theory of color vision. Psychological Review 64: 384–404. 13505974 10.1037/h0041403

[28] Webster MA, Mollon JD (1994) The influence of contrast adaptation on colour appearance. Vision Research 34: 1993–2020. 7941399 10.1016/0042-6989(94)90028-0

[29] Webster MA, Miyahara E, Malkoc G, Raker VE (2000) Variations in normal color vision. I. Cone-opponent axes. Journal of the Optical Society of America A 17: 1535–1544. 10.1364/josaa.17.001535 10975363

[30] Webster MA, Miyahara E, Malkoc G, Raker VE (2000) Variations in normal color vision. II. Unique hues. Journal of the Optical Society of America A 17: 1545–1555. 10.1364/josaa.17.001545 10975364

[31] Webster MA, Mollon JD (1997) Adaptation and the colour statistics of natural images. Vision Research 37: 3283–3298. 9425544 10.1016/s0042-6989(97)00125-9

[32] Webster MA, Mollon JD (1991) Changes in colour appearance following post-receptoral adaptation. Nature 349: 235–238. 1987475 10.1038/349235a0

[33] Hine TJ, McIlhagga WH, Cole GR (2012) From thesholds to colour names: The application of an opponent process model. In: Glenda Andrews and David Neumann (Griffith University Gold Coast A, editor. Beyond the Lab: Applications of Cognitive Research in Memory and Learning. Brisbane, Queensland, Australia: Nova Publishers. pp. 175–195.

[34] Wuerger SM, Atkinson P, Cropper SJ (2005) The cone inputs to the unique hue mechanisms. Vision Research 47: 3210–3223. 10.1016/j.visres.2005.06.016 16087209

[35] Malkoc G, Kingdom FA (2012) Dichoptic difference thresholds for chromatic stimuli. Vision Res 62: 75–83. 22487719 10.1016/j.visres.2012.03.018

[36] Burns SA, Elsner AE, Pokorny J, Smith VC (1984) The Abney effect: chromaticity coordinates of unique and other constant hues. Vision Research 24: 479–489. 6740967 10.1016/0042-6989(84)90045-2

[37] WdWSir Abney (1909) On the Change in Hue of Spectrum Colours by Dilution with White Light. Philosophical Transactions of the Royal of Society London series A 83: 120–127.

[38] Boynton RM (1975) Color, Hue and Wavelength. In: Carterette EC, Friedman MP, editors. Handbook of Perception: Academic Press. pp. 302–347.

[39] Danilova MV, Mollon JD (2012) Cardinal axes are not independent in color discrimination. J Opt Soc Am A Opt Image Sci Vis 29: A157–164. 22330373 10.1364/JOSAA.29.00A157

[40] Danilova MV, Mollon JD (2012) Foveal color perception: minimal thresholds at a boundary between perceptual categories. Vision Res 62: 162–172. 22538222 10.1016/j.visres.2012.04.006

[41] Danilova MV, Mollon JD (2010) Parafoveal color discrimination: a chromaticity locus of enhanced discrimination. J Vis 10: 4 1–9. 10.1167/10.1.4 PMC2875107 20143897

[42] Chichilnisky EJ, Wandell BA (1999) Trichromatic opponent color classification. Vision Res 39: 3444–3458. 10615508 10.1016/s0042-6989(99)00033-4

[43] De Valois RL, De Valois KK, Mahon LE (2000) Contribution of S opponent cells to color appearance. Proc Natl Acad Sci U S A 97: 512–517. 10618449 10.1073/pnas.97.1.512 PMC26694

[44] Berlin B, Kay P (1969) Basic color terms: their universality and evolution. Berkeley, USA: University of California press.

[45] Boynton RM (1988) Color vision. Annu Rev Psychol 39: 69–100. 3278683 10.1146/annurev.ps.39.020188.000441

[46] Boynton RM (1978) Colour in contour and object perception. In: Carterette EC, Friedman BM, editors. Handbook of Perception: Academic Press.

[47] Bornstein MH (1973) Colour vision and Colour naming: A psychophysical hypothesis of of cultural difference. Psychological Bulletin 80: 257–285. 4742311 10.1037/h0034837

[48] Hurvich LM, Jameson D (1955) A quantitative theoretical account of color vision. Trans N Y Acad Sci 18: 33–38. 13274444 10.1111/j.2164-0947.1955.tb00126.x

[49] Danilova MV, Mollon JD (2010) Parafoveal color discrimination: a chromaticity locus of enhanced discrimination. J Vis 10. 10.1167/10.1.4 PMC2875107 20143897

[50] Nosofsky RM (1987) Attention and learning processes in the identification and categorization of integral stimuli. J Exp Psychol Learn Mem Cogn 13: 87–108. 2949055 10.1037//0278-7393.13.1.87

[51] Little DR, Nosofsky R, Donkin C, Denton SE (in press) Logical-rules and the classification of integral dimensioned stimuli. Journal of Experimental Psychology: Learning, Memory & Cognition 10.1037/a0029667 22905932

[52] Nosofsky RM, Palmeri TJ (1997) Comparing exemplar-retrieval and decision-bound models of speeded perceptual classification. Percept Psychophys 59: 1027–1048. 9360476 10.3758/bf03205518

[53] Nosofsky RM, Little DR, Donkin C, Fific M (2011) Short-term memory scanning viewed as exemplar-based categorization. Psychol Rev 118: 280–315. 21355662 10.1037/a0022494 PMC3136045

[54] Nosofsky RM, Little DR, James TW (2012) Activation in the neural network responsible for categorization and recognition reflects parameter changes. Proc Natl Acad Sci U S A 109: 333–338. 22184233 10.1073/pnas.1111304109 PMC3252895

[55] Heit E, Hayes BK (2011) Predicting reasoning from memory. J Exp Psychol Gen 140: 76–101. 21299318 10.1037/a0021488

[56] Heit E, Hayes BK (2005) Relations among categorization, induction, recognition, and similarity: comment on Sloutsky and Fisher (2004). J Exp Psychol Gen 134: 596–605 discussion 606–611. 16316295 10.1037/0096-3445.134.4.596

[57] Heit E, Rotello CM, Hayes BK (2012) Relations between memory and reasoning. In: Ross B, editor. The psychology of learning and motivation. San Diego, CA.: Academic Press.

[58] Cohen AL, Nosofsky RM (2000) An exemplar-retrieval model of speeded same–different judgments. J Exp Psychol Hum Percept Perform 26: 1549–1569. 11039484 10.1037//0096-1523.26.5.1549

[59] Shepard RN, Chang JJ (1963) Stimulus generalization in the learning of classifications. J Exp Psychol 65: 94–102. 13988437 10.1037/h0043732

[60] Fific M, Nosofsky RM, Townsend JT (2008) Information-processing architectures in multidimensional classification: a validation test of the systems factorial technology. J Exp Psychol Hum Percept Perform 34: 356–375. 18377176 10.1037/0096-1523.34.2.356 PMC2650621

[61] Garner WR (1974) The processing of information and structure. Potomac, Md.: LEA.

[62] Garner WR, Felfoldy GL (1970) Integrality of stimulus dimensions in various types of information processing. Cognitive Psychology 1: 225–229.

[63] Shepard RN (1992) The perceptual organization of colors: An adaptation to regularities of the terrestrial world? In: Barkow, Cosmides, Tooby, editors. The Adapted Mind. pp. 495–532.

[64] Goldstone RL (1994) The role of similarity in categorization: providing a groundwork. Cognition 52: 125–157. 7924201 10.1016/0010-0277(94)90065-5

[65] Ozgen E, Davies IRL (2002) Acquisition on categorical colour perception: A perceptual learning approach to the linguistic relativity hypothesis. Journal of Experimental Psychology: General 131: 477–493. 12500859

[66] Indow T (1988) Multidimensional studies of Munsell color solid. Psychol Rev 95: 456–470. 3057527 10.1037/0033-295x.95.4.456

[67] Indow T, Kanazawa K (1960) Multidimensional mapping of Munsell colors varying in hue, chroma, and value. J Exp Psychol 59: 330–336. 13852802 10.1037/h0044796

[68] Indow T, Ohsumi K (1972) Multidimensional mapping of sixty Munsell colors by nonmetric procedure. In: Vos J, et al.., editor. Color Metrics. Holland: AIC.

[69] De Valois RL, De Valois KK (1993) A multi-stage color model. Vision Res 33: 1053–1065. 8506645 10.1016/0042-6989(93)90240-w

[70] De Valois RL, Cottaris NP, Mahon LE, Elfar SD, Wilson JA (2000) Spatial and temporal receptive fields of geniculate and cortical cells and directional selectivity. Vision Res 40: 3685–3702. 11090662 10.1016/s0042-6989(00)00210-8

[71] Cottaris NP, De Valois RL (1998) Temporal dynamics of chromatic tuning in macaque primary visual cortex. Nature 395: 896–900. 9804422 10.1038/27666

[72] Macleod DIA, Boynton RM (1979) Chromaticity diagram showing cone excitation by stimuli of equal luminance. Journal of the Optical Society of America 69: 1183–1186. 490231 10.1364/josa.69.001183

[73] Smith VC, Pokorny J (1975) Spectral sensitivity of the foveal cone photopigments between 400 and 500 nm. Vision Res 15: 161–171. 1129973 10.1016/0042-6989(75)90203-5

[74] Smith VC, Pokorny J (1972) Spectral sensitivity of color-blind observers and the cone photopigments. Vision Res 12: 2059–2071. 4539070 10.1016/0042-6989(72)90058-2

[75] Wyszecki G, Stiles WS (1967) Color Science. New York: John Wiley and Sons.

[76] Cropper SJ (2006) The detection of motion in chromatic stimuli: Pedestals and masks. Vision Research 46: 724–738. 16112703 10.1016/j.visres.2005.06.034

[77] Cropper SJ (2005) The detection of motion in chromatic stimuli: First-order and second-order spatial structure. Vision Research 45: 265–280. 10.1016/j.visres.2004.09.043 15644227

[78] Anstis SM, Cavanagh P (1983) A minimum motion technique for judging equiluminance. In: Mollon JD, Sharpe LT, editors. Colour Vision: Physiology and psychophysics. London: Academic Press. pp. 154–166.

[79] Cavanagh P, Anstis S (1991) The contribution of color to motion in normal and color-deficient observers. Vision Research 31: 2109–2148. 1771796 10.1016/0042-6989(91)90169-6

[80] Wagner G, Boynton RM (1972) A Comparison of four methods of heterochromatic photometry. Journal of the Optical Society of America 62: 1508–11515. 4643012 10.1364/josa.62.001508

[81] Cropper SJ, Mullen KT, Badcock DR (1996) Motion coherence across different chromatic axes. Vision Research 36: 2475–2488. 8917809 10.1016/0042-6989(95)00299-5

[82] Findlay JM (1978) Estimates on probability functions: a more virulent PEST. Perception and Psychophysics 23: 181–185.

[83] Wright O (2011) Effect of stimulus range on colour categorisation. In: Biggam CP, Hough CA, Kay CJ, Simmons DR, editors. New Directions in Colour Science. Amsterdam, NL.: John Benjamin's Publishing. pp. 265–276.

[84] Green DM, Swets JA (1966) Signal detection theory and psychophysics. NY: John Wiley.

[85] Macmillan NA, Creelman CD (1991) Detection theory: A users guide. New York: Cambridge University Press.

[86] Hansen T, Pracejus L, Gegenfurtner KR (2009) Color perception in the intermediate periphery of the visual field. J Vis 9: 26 21–12. 10.1167/9.4.26 19757935

[87] Hansen T, Walter S, Gegenfurtner KR (2007) Effects of spatial and temporal context on color categories and color constancy. J Vis 7: 2. 10.1167/7.4.2 17461686

[88] Cropper SJ, Little DR, Kvansakul JGS (2013) The categorisation of non-categorical colours: Learning a new category. PLOS One In Preparation. 10.1371/journal.pone.0059945 PMC3607564 23536899

[89] Travis D (1991) Effective Color Displays: Theory and Practice. San Diego, CA: Academic Press Inc.

[90] McDermott KC, Webster MA (2012) Uniform color spaces and natural image statistics. Journal of the Optical Society of America A: Optics, Image Science, and Vision 29: A182–A187. 10.1364/JOSAA.29.00A182 PMC3281518 22330376

[91] Wright WD (1928) A re-determination of the trichromatic coefficients of the spectral colours. Transactions of the Optical Society 34: 141–164.

[92] Guild J (1931) The colorimetric properties of the spectrum. Philosophical Transactions of the Royal Society of London series A 230: 149–187.

[93] Shepard RN (1987) Toward a universal law of generalization for psychological science. Science 237: 1317–1323. 3629243 10.1126/science.3629243

[94] Hooke R, Jeeves TA (1961) Direct search solution of numerical and statistical problems. Journal of the ACM 8: 212–229.

[95] Schwarz GE (1978) Estimating the dimension of a model. Annals of Statistics 6: 461–464.

[96] Davidoff J (2001) Language and perceptual categorisation. Trends Cogn Sci 5: 382–387. 11520702 10.1016/s1364-6613(00)01726-5

[97] Davidoff J (1986) The mental representation of faces: spatial and temporal factors. Percept Psychophys 40: 391–400. 3808905 10.3758/bf03208198

[98] Davidoff J, Davies I, Roberson D (1999) Colour categories in a stone-age tribe. Nature 398: 203–204. 10094043 10.1038/18335

[99] Davidoff J, Goldstein J, Tharp I, Wakui E, Fagot J (2012) Perceptual and categorical judgements of colour similarity. Journal of Cognitive Psychology 24: 871–892.

[100] Roberson D, Davies I, Davidoff J (2000) Color categories are not universal: replications and new evidence from a stone-age culture. J Exp Psychol Gen 129: 369–398. 11006906 10.1037//0096-3445.129.3.369

[101] Smithson HE, Khan SS, Sharpe LT, Stockman A (2006) Transitions between color categories mapped with a reverse Stroop task. Vis Neurosci 23: 453–460. 16961980 10.1017/S0952523806233388

[102] Ozgen E (2004) Language, learning, and color perception. Current Directions in Psychological Science 13: 95–98.

[103] Pilling M, Wiggett A, Ozgen E, Davies IRL (2003) Is color “categorical perception” really perceptual? Memory and Cognition 31: 538–551. 12872870 10.3758/bf03196095

[104] Marr D (1982) Vision: A computational investigation into the human representation and processing of visual information. San Francisco: W.H. Freeman and Company.

[105] Lennie P, Krauskopf J, Sclar G (1990) Chromatic mechanisms in striate cortex of macaque. The Journal of Neuroscience 10: 649–669. 2303866 10.1523/JNEUROSCI.10-02-00649.1990 PMC6570166

[106] Horwitz GD, Hass CA (2012) Nonlinear analysis of macaque V1 color tuning reveals cardinal directions for cortical color processing. Nat Neurosci 10.1038/nn.3105 PMC3528341 22581184

[107] Cropper SJ, Wuerger SM (2005) The perception of motion in chromatic stimuli. Behavioural & Cognitive Neuroscience Reviews 4: 192–217. 10.1177/1534582305285120 16510893

[108] Munsell AH (1915) Atlas of the Munsell color system. Malden, MA: Wadsworth-Holland.

[109] Neitz J, Carroll J, Yamauchi Y, Neitz M, Williams DR (2002) Color perception is mediated by a plastic neural mechanism that is adjustable in adults. Neuron 35: 783–792. 12194876 10.1016/s0896-6273(02)00818-8

